# Identifying and characterizing high-risk clusters in a heterogeneous ICU population with deep embedded clustering

**DOI:** 10.1038/s41598-021-91297-x

**Published:** 2021-06-08

**Authors:** José Castela Forte, Galiya Yeshmagambetova, Maureen L. van der Grinten, Bart Hiemstra, Thomas Kaufmann, Ruben J. Eck, Frederik Keus, Anne H. Epema, Marco A. Wiering, Iwan C. C. van der Horst

**Affiliations:** 1 Department of Clinical Pharmacy and Pharmacology, University of Groningen, University Medical Center Groningen, Hanzeplein 1, P.O. Box 30.00, 9700 RB Groningen, The Netherlands; 2 Department of Anesthesiology, University of Groningen, University Medical Center Groningen, Groningen, The Netherlands; 3 Bernoulli Institute for Mathematics, Computer Science and Artificial Intelligence, University of Groningen, Groningen, The Netherlands; 4 Department of Internal Medicine, University of Groningen, University Medical Center Groningen, Groningen, The Netherlands; 5 Department of Critical Care, University of Groningen, University Medical Center Groningen, Groningen, The Netherlands; 6 Department of Intensive Care, Maastricht University Medical Centre+, University Maastricht, Maastricht, The Netherlands

**Keywords:** Medical research, Preclinical research, Diagnostic markers, Prognostic markers, Computer science

## Abstract

Critically ill patients constitute a highly heterogeneous population, with seemingly distinct patients having similar outcomes, and patients with the same admission diagnosis having opposite clinical trajectories. We aimed to develop a machine learning methodology that identifies and provides better characterization of patient clusters at high risk of mortality and kidney injury. We analysed prospectively collected data including co-morbidities, clinical examination, and laboratory parameters from a minimally-selected population of 743 patients admitted to the ICU of a Dutch hospital between 2015 and 2017. We compared four clustering methodologies and trained a classifier to predict and validate cluster membership. The contribution of different variables to the predicted cluster membership was assessed using SHapley Additive exPlanations values. We found that deep embedded clustering yielded better results compared to the traditional clustering algorithms. The best cluster configuration was achieved for 6 clusters. All clusters were clinically recognizable, and differed in in-ICU, 30-day, and 90-day mortality, as well as incidence of acute kidney injury. We identified two high mortality risk clusters with at least 60%, 40%, and 30% increased. ICU, 30-day and 90-day mortality, and a low risk cluster with 25–56% lower mortality risk. This machine learning methodology combining deep embedded clustering and variable importance analysis, which we made publicly available, is a possible solution to challenges previously encountered by clustering analyses in heterogeneous patient populations and may help improve the characterization of risk groups in critical care.

## Introduction

Critically ill patients constitute a highly heterogeneous population, with high rates of acute and chronic multimorbidity, and different profiles of risk, response to interventions, and outcomes. Despite extensive research, however, the goal of unravelling patient heterogeneity remains largely unattained. Critical care clinicians rely on a combination of laboratory and clinical examination variables, and their own clinical experience (clinical gestalt) and gut feeling to characterize patients^1^. While human beings excel at ascribing meaning to observed patterns once they have seen them, data-driven approaches enabling the combination of diverse data streams can enhance patient characterization within known “sub-phenotypes” or provide insight into new categorizations^2,3^.

Clustering analysis has been used for several purposes in medical research, with different approaches showing promising results to help identify and characterize relevant clusters. One of the main appeals of clustering analysis is that its principles resemble a heuristic which clinicians are familiar with: it finds similarities and differences between patients and divides them into group^1^. Within critical care research in particular, latent class analysis (LCA) has been the most broadly used algorithm for sub-phenotype identification in cohorts of patients with acute respiratory distress syndrome (ARDS) and acute kidney injury (AKI)^4,5^*.* LCA is an established, model-based statistical technique, that defines the best fitting models for data assumed to contain several unobserved groups^4,5^. Unlike LCA, where clusters are derived from the distribution of the data, unsupervised clustering algorithms such as k-means and hierarchical clustering find clusters by identifying similarities between cases^1^. These two algorithms have recently been applied to identify clusters in a general ICU population, cardiovascular clusters in septic shock patients, and corticosteroid response in patients with severe asthma, with a variation of k-means called fuzzy c-means also being used to cluster severely injured blunt trauma patients^2,6–8^.

Similarly, prediction models with increasingly higher accuracy and explainability for mortality and organ injury have been suggested, capable of processing high-frequency data^9–11^. Both approaches have different advantages and shortcomings. Prediction models can deal with virtually any data format and provide individual probabilities for an outcome, but are bound by a priori hypotheses and can only compute the probability of one specific outcome. On the other hand, traditional clustering algorithms, are not designed to process the high-frequency, dynamic data collected in ICU^1^.

In this study, we sought to develop and apply a novel approach to identifying and characterizing clusters of critically ill patients. Using co-morbidity, clinical examination, and laboratory data from a minimally selected ICU cohort, we compared the performance of different clustering methodologies and applied a combined deep embedded clustering and feature importance analysis algorithm to identify clusters of patients at high risk of AKI and mortality during ICU stay, and at 30 and 90 days. Then, we trained a classifier to predict and validate cluster membership, and identified the features driving these predictions. We hypothesized that this approach could identify clinically recognizable patient clusters with clinically significant differences in mortality and severe acute kidney injury.

## Methods

### Data sources

Data used for this study originated from the prospective, single-centre Simple Intensive Care Studies (SICS) I cohort study. All acutely admitted, critically ill patients included the study underwent clinical examination and critical care ultrasonography (CCUS) within the first 24 h of ICU admission. Informed consent was obtained for all included patients, and all analyses were performed in accordance with relevant guidelines and regulations. Further details on inclusion criteria, informed consent, and study protocol are available elsewhere^12,13^. The study was approved by the local institutional review board (Medisch Ethische Toetsingscommissie (METc) of the UMCG, M15.168207).

### Co-morbidity, clinical examination, and laboratory data

The dataset consisted of patient characteristics including co-morbidities, clinical examination variables including CCUS, vital signs, and urine output, and a time-series of 40 laboratory values measured at least once daily (Table 1). CCUS measurements were validated by experts, and vital signs were recorded from the bedside monitor^12,13^. Patients with more than 10% missing data (i.e. variables for which no measurements were registered at any moment during ICU stay) were excluded from the analysis. Missing laboratory data were imputed using a rolling mean based on ICU-specific values^14^. For other variables, iterative imputation with 10 iterations (a method similar to multivariate imputation by chained equations) was used^15^.

**Table 1:** Complete list of input features. List including all patient characteristics, co-morbidities, time-series of laboratory parameters, and clinical examination parameters.

Patient characteristics	Age, sex, APACHE IV Score, SAPS II Score, BMI, surgical admission, previous admission to ICU
Clinical examination	**Hemodynamic parameters** Cardiac index, mottling, atrial fibrillation, heart rate at admission, urine output in previous 6 h, prolonged capillary refill time, central venous pressure (CVP), diastolic blood pressure (DBP), systolic blood pressure (SBP), mean arterial pressure (MAP) **Respiratory parameters** Worsened respiratory condition after 24 h assessed by physician, tidal volume, Respiratory rate of ventilator, positive end-expiratory pressure (PEEP) of ventilator, mechanical ventilation after 24 h (binary), mechanical ventilation at admission (binary), respiratory rate, lowest FiO2 (%) during ICU stay **Other** EMV score
Co-morbidities and medical history	History of cardiovascular disease (CVD), history of chronic kidney disease (CKD), history of cirrhosis, history of chronic obstructive pulmonary disease (COPD), history of diabetes, history of hematological malignancy, history of metastatic disease, history of myocardial infarction, history of respiratory insufficiency, history of acquired immunodeficiency syndrome (AIDS), history of immune insufficiency, previous dialysis
Laboratory variables (for which the mean and variance were taken)	**Routinely collected** ALAT, ASAT, albumin, amylase, ALP, bilirubin (total), gamma-GT, CK, CRP, calcium, chloride, magnesium, MCV, sodium, phosphate, potassium, fibrinogen, hemoglobin, hematocrit, creatinine, LDH, leukocytes, thrombocytes, troponin T, total protein, urea **Arterial POC** Ionized calcium, glucose, hemoglobin, potassium, lactate, sodium, arterial HCO_3_, arterial pCO_2_, arterial pH, arterial pO_2_, arterial saturation, methylated hemoglobin, HbCO

*ALAT* alanine transaminase, *ASAT* aspartate transaminase, *CK* creatine kinase, *CRP* C-reactive protein, *LDH* lactate dehydrogenase, *POC* point of care, *HCO*_*3*_ bicarbonate, *pCO*_*2*_ arterial CO_2_ pressure, *pO*_*2*_ arterial O_2_ pressure, *HbMet* methemoglobin, *HbCO* carboxyhemoglobin.

Feature extraction (mean and variance concatenated over the whole time-series) was employed to represent the time-series data. Table 1 shows the average number of co-morbid conditions per patient per cluster, calculated based on the information on co-morbidities.

### Outcome

To define and assess clinically relevant differences between clusters, mortality at three time points (in-ICU, as well as at 30 and 90 days) was taken as a primary outcome. Kaplan Meier curves were used to visualize the mortality per cluster during and after ICU stay. The secondary outcome was the development of severe AKI (stages 2 or 3). Additionally, differences in the development of any stage of AKI, need for vasopressors, ICU length of stay, development of shock, and need for renal replacement therapy are also reported.

### Development and comparison of different clustering methodologies

Most clustering algorithms, such as k-means clustering and hierarchical clustering, are not designed to process high-frequency, dynamic data^1^. Different strategies have been developed to facilitate this, including combining K-means and HC with a time-series processing methodology such as dynamic time warping (DTW), as well as using clustering algorithms which represent data in a different way, such as deep embedding clustering (DEC)^16,17^. In both these approaches, described in more detail in the Supplementary information files, features such as mean and variance are extracted from the time-series data and subsequently fed into a clustering algorithm. In this study, we compared a DEC model to two “traditional” clustering algorithms, k-means and hierarchical clustering (HC), as well as a combination of HC and dynamic time warping (HC-DTW).

Deep embedded clustering algorithms utilize autoencoder neural networks to learn a certain representation of the data, and then use this representation to form clusters^16^. Despite its frequent use in for clustering analyses in other fields, there are but a few reports of analysis of medical data using DEC^18^. The DEC model developed in this study combined a multilayer perceptron (MLP) autoencoder, which is a type of neural network, and a custom clustering layer with the k-means clustering algorithm. The clustering layer reconstructs features created by the MLP autoencoder, and converts it to cluster label probabilities represented by Student’s t-distribution. The clustering layer weights represent the cluster centroids and are initialized using k-means algorithm. To improve cluster purity, a centroid-based target distribution is constructed by squaring the encoded vectors and normalizing them by frequency per cluster. Finally, the algorithm is trained to minimize Kullback–Leibler divergence loss for a maximum of 8000 iterations with 0.01 tolerance threshold.

Once clusters were computed for all four algorithms, validity assessments were conducted^19^. Internal validity assesses whether the structure of the clustering is intrinsically appropriate for the data. Patients clustered in the same cluster should have similar data, whereas patients from different clusters should be as distinct as possible from those in other clusters. Here, the Silhouette index was used to internally validate k-means, HC, and HC-DTW. For DEC, cluster-wise stability was computed by resampling the dataset 100 times and computing the Jaccard similarities to the originally defined clusters as well as entropy scores^20–22^. External validity assesses whether clustering results match some a priori expected data structure. When the true cluster labels are known, this is done by comparing the clustering output to a given “correct” clustering^1,23^. Since no “true” labels are available when attempting to identify new putative patient clusters, the clinical recognizability of these clusters was used as surrogate of external validity. Lastly, to compare the different methodologies, the potential clinical utility of the clustering was assessed by examining the distribution of patients across the different clusters and whether the different clustering configurations identified between-group differences in the input features.

### Cluster membership prediction and feature importance analysis for cluster characterization

**Figure 1:**
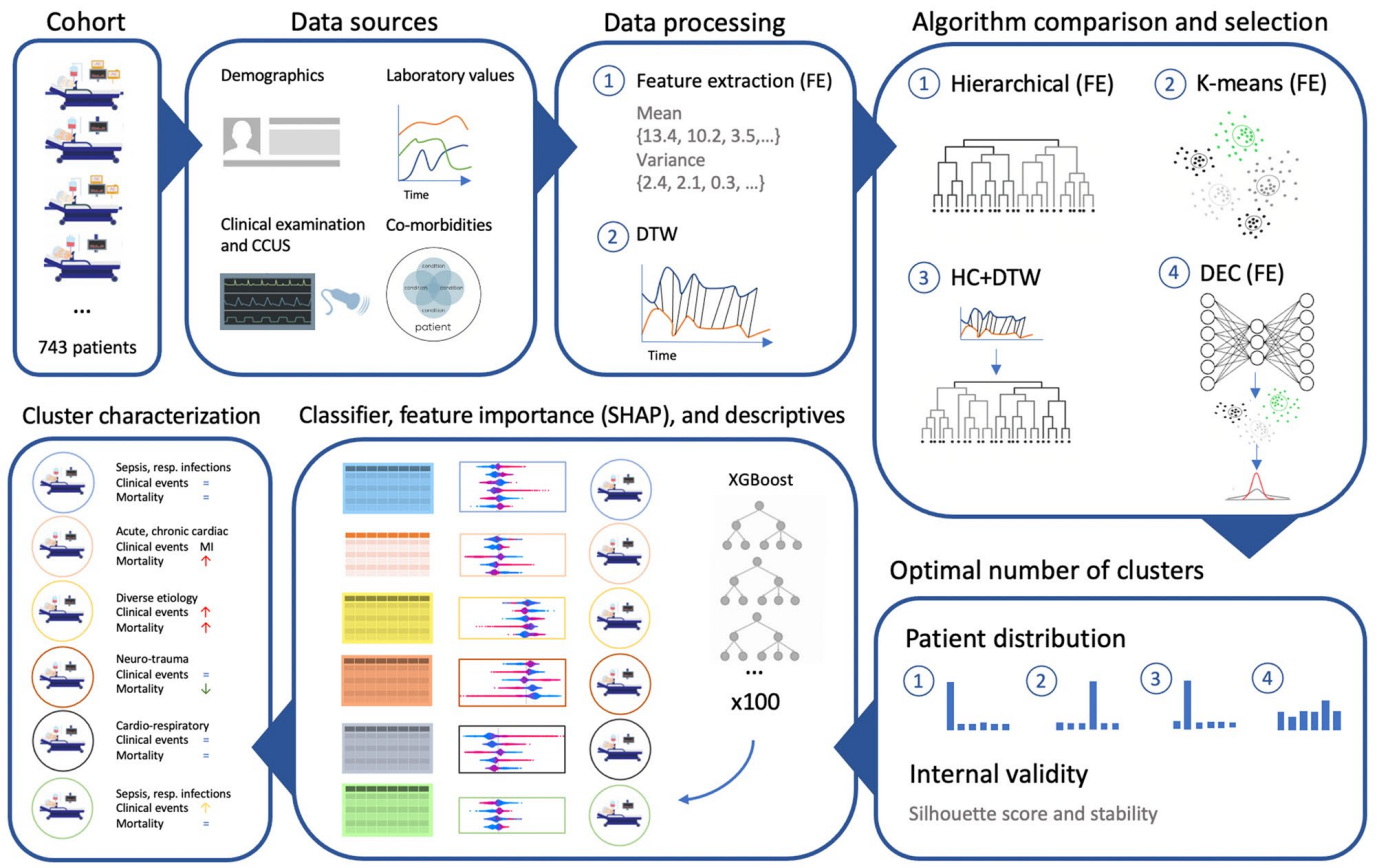
Schematic overview of the different steps in the analysis. Patient selection, integration of different data sources, data processing with feature extraction (FE) or dynamic time warping (DTW), comparison of the four clustering algorithms, selection of the best algorithm based on patient distribution and internal validity measures, training of the classifier for attributing true labels to the clusters and calculating feature importance with SHAP, and cluster characterization based on input data from diagnoses, feature importance, and differences in outcomes including mortality, AKI, and other clinical events.

A gradient boosting algorithm (XGBoost) was trained to predict cluster membership over 10-folds for each of the 100 clustering configurations resulting from DEC^24^. Then, SHapley Additive exPlanations (SHAP) values were computed on the run with the highest accuracy to represent the feature importance of each variable in the model. SHAP values are widely used in game theory to determine the contribution of particular features to the difference between the actual and the mean predictions^25^. Positive and negative SHAP values signal that variables contribute positively or negatively to cluster membership, respectively. Finally, clusters were characterized based on the between-cluster differences in input variables and outcomes, and feature importance values. The admission and discharge diagnoses of all patients were analysed and relevant clinical information to aid in the characterization was extracted. A full schematic overview of the analysis is provided in Fig. 1.

### Statistical analysis

Descriptive characteristics for the study population were reported as means with standard deviations and proportions for continuous and categorical variables, respectively. Differences in input variables and outcomes between clusters were determined using analysis of variance and chi-squared tests. Hazard ratios for mortality per cluster were computed, and the *p*-value for comparison against the full cohort was calculated using the log-rank test. A *p*-value below 0.05 was considered significant. All clustering and further statistical analyses were performed using Python with PyCharm as interface (version 2019.3.5).

## Results

### Study population and outcome

Of the 1075 patients included in SICS-I, 743 had less than 10% missing data and were included in the analysis. Both the numbers of variables and the numbers of measurements per variable varied per patient, with on average 21 measurements of each variable per patient. The average number of measurements per variable per patient, and the average time between measurements for each variable across all patients are presented in Supplementary Table S2. Patient characteristics, clinical examination variables, and outcomes are reported in Table 2. Complete data on laboratory variables and outcomes can be found in Supplementary Tables S3–S6, and Supplementary Figs. S9–S10. After 30 and 90 days, 166 and 205 patients (22.3%) had died, with no patients lost to follow-up.

**Table 2:** Clinical characteristics for the 743 patients, including patient characteristics, clinical examination data, co-morbidities and medical history, and outcomes.

**Patient characteristics**	
Age (years)	62 [61, 63]
Gender (% female)	63.3
APACHE IV Score	76.9 [74.8, 79.2]
SAPS II Score	46.9 [45.7, 48.0]
BMI	26.7 [26.4, 27.1]
Surgical patient (%)	35.0
Previous admission to ICU (%)	11.6
Clinical examination	
Cardiac index > 2.2 (%)	42.7
Mottling (% with severe, > 4)	3.1
Mechanically ventilated at admission (%)	61.8
Worsened respiratory condition after 24 h (%)	12.4
Urine output in previous 6 h (ml/kg/h)	0.9 [0.84, 0.95]
CRT prolonged (%)	29.9
EMV score	11.27 [10.9, 11.63]
**Co-morbidities and medical history**	
History of CVD (%)	5.0
History of CKD (%)	6.9
History of cirrhosis (%)	3.4
History of COPD (%)	12.4
Previous dialysis (%)	1.4
History of diabetes (%)	20.3
History of hematological malignancy (%)	4.0
History of metastatic disease (%)	3.6
History of myocardial infarction (%)	8.2
History of respiratory failure (%)	4.9
**Admission diagnosis by organ system**^a^	
Cardiovascular	32.8
Gastrointestinal	14.2
Genito-urinary	1.2
Haematological	1.4
Metabolic	2.3
Musculoskeletal/skin	0.9
Neurological	15.0
Respiratory	19.5
Transplant	5.1
Trauma	7.7
**Outcomes**^a^	
In-ICU mortality (%)	19.4
30-day mortality (%)	22.3
90-day mortality (%)	27.6
No AKI (%)	79.7
AKI stage 1 (%)	59.8
AKI stage 2 or 3 (%)	44.3
Required vasoactive medication (%)	48.6
Any type of shock (%)	51.6
Required RRT (%)	10.1
ICU length of stay (days)	6.0 [5.4, 6.6]

^a^Denotes variables not included as input for clustering analysis.

### Performance of different clustering methodologies

Across k-means, HC, and HC-DTW, the silhouette score was highest when patients were divided into only 2 clusters (Supplementary Table S1). When 3–6 clusters were considered, silhouette scores were still similar, but lower, across algorithms. However, the high silhouette scores are explained by all three algorithms always grouping most patients into one of the clusters, even when the dimensionality (and hence the noise) of the inputs was reduced using principal component analysis. As shown in Supplementary Table S1, all three methods tended to cluster around 90% of patients in only one cluster, regardless of the number of clusters. In contrast, clusters generated by DEC showed balanced cluster membership distribution irrespective of the putative number of clusters (Supplementary Table S1) and identified significant between-cluster differences for the majority of the input features (Supplementary Tables S3–S5). Amongst the seven different possible clustering configurations generated by DEC, stability was highest for six clusters (Supplementary Figs. S1–S8). The tenfold cross-validation XGBoost model predicted cluster membership with 83% accuracy, with sensitivity ranging from 64 to 90% and specificity from 85 to 100% (Supplementary Table S7, Supplementary Fig. S11).

### Feature importance analysis and cluster characterization

**Figure 2:**
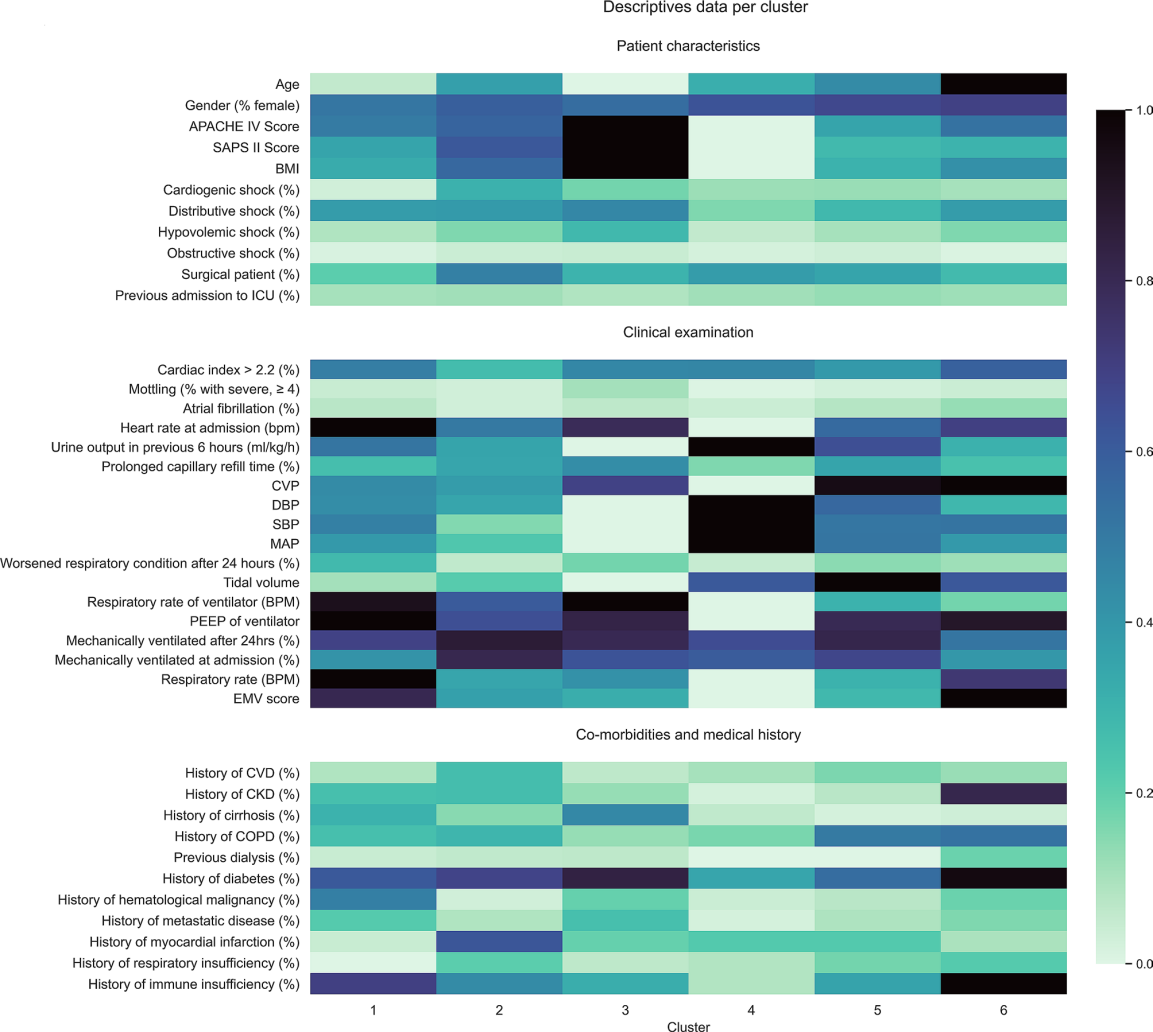
Heatmap of patient characteristics, clinical examination and co-morbidity data per cluster. Bars on the right show the colour scale representing the proportion of patients with each characteristic regarding demographics, clinical examination, and co-morbidities. For continuous variables, such as SBP or urine output, it represents a scaled value from highest cluster mean (1.0) to lowest cluster mean (0.0).

**Figure 3:**
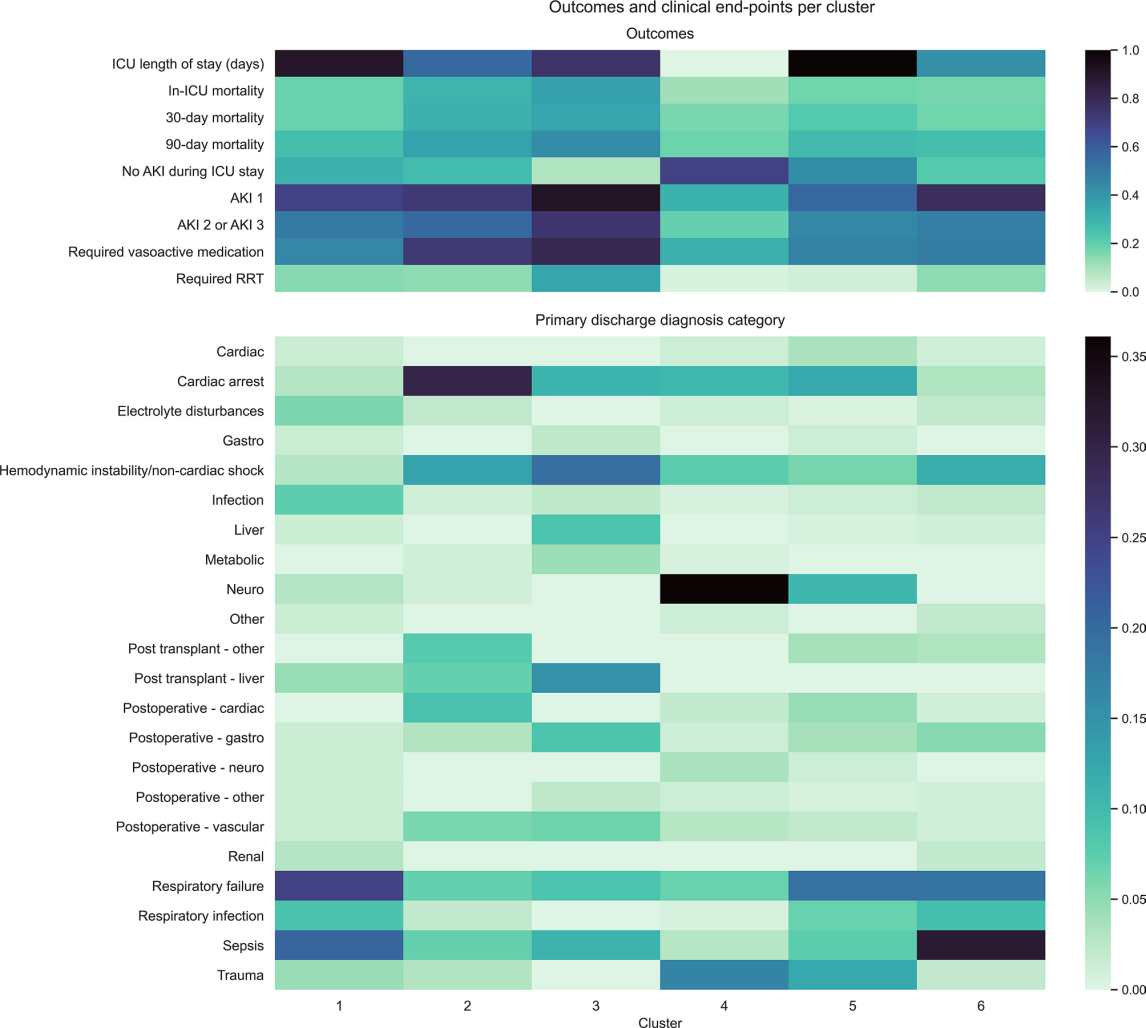
Heatmap of outcomes and clinical end-points per cluster. Bars on the right show the colour scale representing the proportion of patients within the cluster with the outcome (upper panel) or the discharge diagnosis (lower panel).

**Figure 4:**
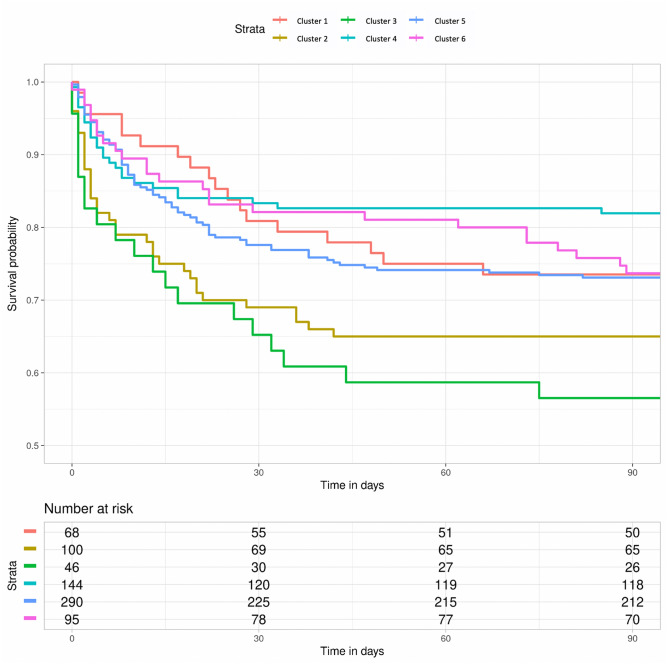
Kaplan–Meier curves stratified per cluster for mortality during and after ICU stay. Survival curves for all six clusters, with the number of patients at risk at 30 and 90 days per cluster.

Sixty-eight patients with high prevalence of respiratory failure or infection (34%), as well as sepsis (21%), were assigned to cluster 1 (Fig. 3, Supplementary Table S3). These patients had a long ICU stay, and the highest rate of worsened respiratory condition after 24 h (Fig. 2, Supplementary Tables S3–S5 and Supplementary Fig. S9). Feature importance analysis identified increased alkaline phosphatase, gamma-GT, bilirubin and lactate as having the greatest impact on cluster membership predictions (Supplementary Fig. S12). Higher values for the former three variables drove predictions towards cluster membership, while a high lactate was associated with non-membership. Patients in this cluster were not at increased mortality risk (ICU, 30-day or 90-day; Table 3 and Fig. 4).

**Table 3:** Mortality rates, and hazard ratios for mortality and acute kidney injury per cluster.

Outcome/cluster	%	Hazard ratio with 95%CI	p-value
In-ICU Mortality	%		
Cluster 1	19.1	0.99 [0.77–1.26]	0.916
Cluster 2	30.0	1.55 [1.26–1.91]	< 0.001
Cluster 3	34.8	1.80 [1.33–2.42]	< 0.001
Cluster 4	11.1	0.57 [0.48–0.68]	< 0.001
Cluster 5	17.9	0.92 [0.81,1.06]	0.254
Cluster 6	16.8	0.87 [0.70,1.07]	0.191
**30-day mortality**			
Cluster 1	19.1	0.85 [0.67–1.10]	0.218
Cluster 2	31.0	1.39 [1.12–1.71]	0.002
Cluster 3	37.0	1.66 [1.23–2.23]	< 0.001
Cluster 4	16.7	0.75 [0.63–0.89]	0.001
Cluster 5	22.4	1.00 [0.86–1.15]	0.972
Cluster 6	17.9	0.80 [0.65–0.99]	0.042
**90-day mortality**			
Cluster 1	26.5	0.96 [0.75–1.23]	0.763
Cluster 2	36.0	1.30 [1.06–1.61]	0.012
Cluster 3	43.5	1.58 [1.17–2.12]	0.002
Cluster 4	18.1	0.66 [0.55–0.78]	< 0.001
Cluster 5	27.6	1.00 [0.88–1.15]	0.996
Cluster 6	26.3	0.95 [0.77–1.18]	0.673
**AKI stage 2 or 3**			
Cluster 1	50	1,13 [0.88–1.45]	0.343
Cluster 2	56	1.26 [1.03–1.59]	0.027
Cluster 3	73.9	1.67 [1.24–2.25]	< 0.001
Cluster 4	19.4	0.44 [0.37–0.52]	< 0.001
Cluster 5	45.2	1.02 [0.89–1.17]	0.780
Cluster 6	48.4	1.09 [0.88–1.35]	0.421

Hazard ratios were compared using the log-rank test, with the full cohort used as reference.

*AKI* acute kidney injury.

Cluster 2 (n = 100) included the highest percentage of surgical patients (48%, Fig. 2, Supplementary Table S3), including the largest post-transplant group, and cardiac and vascular procedures (Fig. 3, Supplementary Table S6). Almost 40% of patients presented with acute or chronic cardiac condition, with 63% having a low cardiac index (Fig. 3, Supplementary Table S6). Accordingly, troponin T, lactate dehydrogenase (LDH), creatine kinase (CK) and inflammatory variables were increased (Fig. 2, Supplementary Table S4, Supplementary Fig. S9). Patients in this cluster had higher mortality rates (ICU, 30-day, and 90-day; HR 1.55 [95% CI 1.26–1.91], 1.39 [1.12–1.71], and 1.30 [1.06–1.61], respectively) and were at increased risk of stage 2 or 3 acute kidney injury (HR 1.26 [95% CI 1.03–1.59]) (Fig. 3, Table 3, Supplementary Table S6). Higher values for arterial oxygen (pO_2_), LDH, lactate, troponin and calcium were associated with cluster membership, while low pO_2_, LDH, and ASAT values drove predictions towards non-membership (Supplementary Fig. S13).

Cluster 3 (n = 46) consisted of patients with diverse disease etiology, from liver disease and transplant (24%), to cardiac arrest (11%), sepsis (10%) and respiratory failure (10%). These patients were the youngest and had the highest severity scores at admission (Fig. 2, Supplementary Table S3). Like cluster 2, laboratory variables showed significant elevated troponin T, LDH, CK and inflammatory values (Fig. 2, Supplementary Table S4 and Supplementary Fig. S10). During clinical examination, this group recorded the lowest urine output values (0.58 ml/kg/h), 43% had delayed capillary refill time, and 11% had severe mottling (Fig. 2, Supplementary Table S3). Almost 35% of patients required renal replacement therapy (RRT), and 91% developed AKI, with a HR of stage 2 or 3 AKI of 1.67 [95% CI 1.24–2.25]. These patients also needed the highest vasopressor dose, and had the highest mortality (ICU HR 1.80 [1.33–2.42], 30-day HR 1.66 [1.23–2.23], and 90-day HR 1.58 [1.17–2.12]) (Table 3, Fig. 4). High liver enzymes, LDH, and troponin drove predictions towards cluster membership (Supplementary Fig. S14).

One-hundred forty-four patients with low co-morbidity were in cluster 4, having been admitted primarily with neurosurgical or neurological (40%) and trauma-related (17%) diagnoses (Fig. 3, Supplementary Table S6). These patients presented with relatively lower APACHE IV and SAPS II scores (62 and 41), and had the shortest ICU stay (2.8 days), lowest AKI incidence (31%), and significantly lower risk of mortality (ICU, 30-day, and 90-day (HR 0.57 [0.48–0.68], 0.75 [0.63–0.89], 0.66 [0.55–0.78], respectively). This group also had a markedly reduced risk of severe AKI (HR 0.44 [95% CI 0.37–0.52]). High albumin, hemoglobin, and pO_2_ values predicted membership, while high fibrinogen and potassium predicted non-membership (Supplementary Fig. S15).

For cluster 5 (n = 290), the main admission diagnoses were respiratory failure (19%), cardiac arrest (12%), neurological causes (11%), or trauma (12%). Patients in this cluster had a medium co-morbidity profile, the longest mean ICU stay (7.7 days), and high rates of delayed CRT at admission (35%). Worsening respiratory condition was frequent (14%), and COPD was a common co-morbidity (17%). High thrombocytes and potassium predicted cluster membership, while high values for pO_2_, creatinine, bilirubin, and phosphate drove predictions towards non-membership for some patients (Supplementary Fig. S16). The 95 patients assigned to cluster 6 primarily suffered from sepsis or respiratory infection, having been admitted to the ICU with respiratory (41%) or gastrointestinal diagnoses (20%). They also had high rates of distributive shock (38%), with physical examination showing a high cardiac index in 59% of patients, as well as high respiratory rates and heart rates. High values for fibrinogen, creatinine, urea, and CRP drove predictions towards cluster membership (Supplementary Fig. S17). Patients in these clusters were not at increased or reduced risk of mortality (Table 3).

## Discussion

In this study, we set out to identify patient sub-phenotypes of clinical relevance using time-series laboratory, co-morbidity and clinical examination data. To do this, we compared four different clustering approaches. With a deep clustering algorithm, we identified six sub-phenotypes that capture differences in morbidity and in commonly measured clinical variables. In addition, these sub-phenotypes differed significantly in relevant clinical events rates (such as need for RRT and use of vasopressors) as well as mortality, with one group at low mortality risk, and two at higher mortality risk compared to average.

As the first study evaluating the combination of clustering such a broad range of ICU data including demographic, co-morbidity, clinical examination, and laboratory data with feature importance analysis to characterize patient sub-phenotypes in a minimally-selected ICU population, we draw several conclusions.

We found that traditional clustering algorithms such as k-means and HC were highly susceptible to the variation in data and outliers generated by the inclusion of a large number of laboratory variables. Previous studies using these algorithms with other, mostly low-dimensional datasets had not reported this issue, and do not show the large imbalance in cluster size we observed for k-means and HC in this study^2,6,7^. Interestingly, we identified the same issue with HC-DTW despite the use of a computationally-expensive technique like DTW to make time-series sequences of different length more uniform, and therefore suitable for clustering. Deep embedded clustering, on the other hand, provided a balanced patient distribution across clusters using the extracted features alone. Despite the large amount of data and the moderate cohort size, we managed to achieve stable clusters, and use these labels to train a classifier to identify the features driving cluster membership predictions.

The findings from variable importance analysis provided interesting, adjuvant data for the interpretation of the clusters identified during this analysis. Previous clustering analyses have based interpretation of the phenotypes found on differences in means of the variables measured, or by listing variables related to each cluster based on relative importance^2,6,7^. Here, we complemented the descriptive statistics of each cluster with SHAP values to establish the directionality of the association between high and low values of a variable and cluster membership. For example, membership of clusters with higher mortality risk was associated with increased ASAT and LDH, which are known biomarkers of myocardial ischemia and have also been shown to be positively correlated with ICU mortality^14,26–29^. Likewise, patients were more likely to be in the cluster with the lowest mortality risk (cluster 4) when their albumin, hemoglobin, and pO_2_ values were higher, as well as when their liver function was better. These associations are supported by literature, including the addition of albumin measurements to APACHE scores^30–33^.

The results of our study suggest that this clustering methodology is superior to most widely used approaches for clustering of critically ill patients for several reasons. Firstly, it can process time-series data, unlike k-means, HC, or even HC with dynamic time warping. A recent study using clustering analysis to define cardiovascular phenotypes suggested that incorporating serial measures to study transitions from one phenotype to another during ICU stay would provide additional insight to their analysis, which was limited to static variables^6^. Similarly, clustering studies on treatment response in critically ill patients would benefit substantially from processing time-series data, as opposed to one-time treatment administration^7^.

Secondly, departing from a “minimally-selected” patient cohort, we identified six clusters which differed significantly in mortality and AKI risk, and were also clinically recognizable and describable. Caution has been advised when interpreting the results of clustering analyses, especially when identifying “novel” sub-phenotypes, and rightfully so^3^. Clustering algorithms will inevitably partition patients into clusters, and, as with most unsupervised machine learning techniques, it remains hard to establish what variables drove this partition. It was with this in mind that we set out to cluster patients using a methodology which could identify clusters with significantly different outcomes and simultaneously provided some validation of the results and additional insight into the variables associated with each cluster. This pipeline of clustering, training a classifier on the stable cluster labels, and extraction of SHAP values therefore helped “open the black box” and characterize clusters in more detail.

Lastly, the inclusion of time-series data into clustering analyses can bring a wide range of benefits to studies aiming to characterize patient sub-phenotypes. It can enable the use of continuous hemodynamic monitoring and laboratory data to detect variations in sensitivity to myocardial ischemia or acute kidney injury, or to identify groups with differential treatment responses over time. From a clinical perspective, the potential of accurate clustering of ICU patients to improving patient care is two-fold: it can contribute to better clinical trial design, and it has the potential to inform clinical monitoring and prognostication at bedside. The former idea is supported by recent research on ARDS that suggests sub-phenotyping based on biological markers (such as laboratory parameters) has the potential to identify mechanistic markers proximal to the clinical expression of critical disease and syndromes^34^. By doing so, it can provide a more accurate alternative to clinical phenotyping, which is prone to misclassification and remains challenging even for well-known syndromes, as well as better predict treatment response^34^. Given the challenges critical care trials often face to demonstrate meaningful clinical effects, improved randomization/allocation and design in clinical trials is especially important^35^. Secondly, the identification of clinically meaningful clusters of ICU patients, separating patients by mechanism (cardiac, respiratory, infectious, or other) as well as prognosis (low vs high risk), can be leveraged from ICU admission and throughout ICU stay to guide and optimize staffing tasks. This would allow for not only more personalized care, for example, by minimizing staff contact with patients identified as belonging to clusters with “lower care needs”. In addition, accurate clustering could also be an effective way of summarizing the status of the patient, and could potentially be translated to a (triage) system possibly integrated in a clinical dashboard providing an overview of patients for rapid review by physicians. Naturally, it is essential to replicate and validate the findings of this study before any of these steps can be taken. To this end, we have made the code used in this study publicly available at https://github.com/J1C4F8/SICS_DEC and are currently setting up a multicenter study to assess the validity of the clusters and the feasibility of their clinical application.

Our study also included some limitations. First, while the goal of the SICS-I study was to collect data from a minimally-selected clinical population, inclusion criteria did apply which may account for some selection bias^12,13^. For example, patients expected to stay in the ICU for less than 24 h, due to discharge or extremely dire prognosis, were not included. Second, the six sub-phenotypes identified do not represent an exhaustive classification of critically ill patient subtypes. Information user in previous studies like end-of-life desires, need for life-sustaining therapies, and post-discharge care needs would complement our analysis, which did not include any variables of the disease course after the ICU except for mortality^2^. Third, the SHAP values reported are from an intermediate XGBoost which, despite its moderately high accuracy, does not guarantee the variables identified by SHAP are the exact same variables that the DEC model relied on when creating the clusters. Lastly, external validation of the six identified clusters in an independent cohort is necessary. Future studies with larger datasets should look to validate and replicate our findings, and address the possibility of patients belonging to multiple clusters or whether the addition of other features from the time-series data, such as trend or seasonality, would improve the results we obtained by extracting only the mean and variance of each variable.

In conclusion, our analysis of a cohort with 743 ICU patients, based on a combination of clustering and feature importance analysis of co-morbidity, clinical examination, and laboratory data identified six patient sub-phenotypes with varying mortality and risk of severe acute kidney injury. This machine learning methodology, which we made publicly available, is a possible solution to challenges previously encountered by clustering analyses in heterogeneous populations, and may help improve the characterization of risk groups in critical care.

## Supplementary Information

Supplementary Information.

## Data Availability

The datasets generated during and/or analysed during the current study are not publicly available due to containing sensitive patient information (in particular the detailed admission and discharge diagnoses information) but are available for researchers who meet the criteria for access to confidential data on reasonable request. The code used to create the models is open and available on GitHub, at https://github.com/J1C4F8/SICS_DEC.
