# Supplementary material for: Identifying and characterizing high-risk clusters in a heterogeneous ICU population with deep embedded clustering
Source: Sci Rep. 2021 Jun 8;11:12109. doi: 10.1038/s41598-021-91297-x (PMC8187398; doi:10.1038/s41598-021-91297-x)
Supplement: Supplementary file 1 — Supplementary Information. [file 41598_2021_91297_MOESM1_ESM.pdf]

Identifying and characterizing high-risk clusters in a heterogeneous ICU population with deep embedded clustering

Supplementary material

José Castela Forte BSc (Hon) [1,2,3] (ORCID ID: 0000-0001-9273-0702), Galiya Yeshmagambetova BSc[3], Maureen L. van der Grinten BSc[3], Bart Hiemstra M.D.,Ph.D.[2], Thomas Kaufmann, M.D., Ph.D. [2], Ruben J. Eck M.D.[4], Frederik Keus M.D.,Ph.D. [5], Anne H. Epema M.D.,Ph.D.[2], Marco A. Wiering Ph.D.[3], Iwan C.C. van der Horst M.D.,Ph.D.[6]

Table S1. Silhouette scores, cluster partitioning, and differences in outcomes between different clustering configurations for k-means, hierarchical, and deep embedded clustering. Internal validity assessment was conducted for k-means and hierarchical clustering (HC) by means of Silhouette scores. These indices combine multiple measures (compactness, connectedness, and separation) to provide an estimate of whether the structure of the clustering is appropriate for the data. For clustering algorithms like HC and especially k-means, where defined clusters tend to be spherical, silhouette scores can determine validity well [4]. However, these indices are less useful for analyses on high-dimensional feature spaces, and when compact and clearly separated clusters are not expected, such as is the case within heterogenous critically ill patient populations. For clustering techniques like Deep Embedded Clustering (DEC), cluster stability can be used as an alternative [5,6]. This measure represents the cluster variation over different sub-samples of the same input data and is determined by comparing changes in clusters composition over multiple runs.

	Number of clusters						
	**2**	**3**	**4**	**5**	**6**	**7**	**8**
**K-means without PCA**							
Silhouette score	0.93	0.73	0.73	0.74	0.75	0.75	0.61
Cluster partitioning	1: 732<br>2: 11	1: 708<br>2: 24<br>3: 11	1: 704<br>2: 1<br>3: 20<br>4: 18	1: 700<br>2: 16<br>3: 4<br>4: 1<br>5: 22	1: 689<br>2: 24<br>3: 20<br>4: 5<br>5: 4<br>6: 1	1: 670<br>2: 5<br>3: 1<br>4: 10<br>5: 24<br>6: 29<br>7: 4	1: 661<br>2: 5<br>3: 4<br>4: 22<br>5: 10<br>6: 1<br>7: 32<br>8: 8
In-ICU mortality differences	No	No	No	No	No	No	No
30-day mortality differences	No	No	No	No	No	No	No
90-day mortality differences	No	No	No	No	No	No	No
**K-means with PCA**							
Silhouette score	0.84	0.72	0.75	0.68	0.69	0.57	0.66
Cluster partitioning	1: 732<br>2: 11	1: 695<br>2: 42<br>3: 6	1: 702<br>2: 24<br>3: 16<br>4: 1	1: 679<br>2: 9<br>3: 19<br>4: 1<br>5: 35	1: 689<br>2: 5<br>3: 20<br>4: 24<br>5: 1<br>6: 4	1: 9<br>2: 649<br>3: 10<br>4: 1<br>5: 10<br>6: 4<br>7: 60	1: 669<br>2: 22<br>3: 1<br>4: 2<br>5: 4<br>6: 32<br>7: 8<br>8: 5
In-ICU mortality differences	No	No	No	No	No	No	No
30-day mortality differences	No	No	No	No	No	No	No
90-day mortality differences	No	No	No	No	No	No	No
**HC**							
Silhouette score	0.84	0.78	0.78	0.79	0.62	0.63	0.61
Cluster partitioning	1: 742<br>2: 1	1: 11<br>2: 1<br>3: 731	1: 731<br>2: 7<br>3: 4<br>4: 1	1: 729<br>2: 7<br>3: 4<br>4: 1<br>5: 2	1: 4<br>2: 7<br>3: 692<br>4: 1<br>5: 2<br>6: 37	1: 692<br>2: 7<br>3: 37<br>4: 1<br>5: 2<br>6: 3<br>7: 1	1: 691<br>2: 7<br>3: 37<br>4: 1<br>5: 2<br>6: 3<br>7: 1<br>8: 1
In-ICU mortality differences	No	No	No	No	No	No	No
30-day mortality differences	No	No	No	No	No	No	No
90-day mortality differences	No	No	No	No	No	No	No
**HC-DTW**							
Silhouette score	0.87	0.86	0.58	0.59	0.59	0.52	N/A
Cluster partitioning	1:742<br>2:1	1:740<br>2:1	1:714<br>2:26	1:708<br>2:26	1:26<br>2:706	1:706<br>2:22	N/A

		3:2	3:2<br>4:1	3:2<br>4:1<br>5:6	3:2<br>4:1<br>5:6<br>6:2	3:2<br>4:1<br>5:6<br>6:2<br>7:4	
In-ICU mortality differences	No	No	No	No	No	No	N/A
30-day mortality differences	No	No	No	No	No	No	N/A
90-day mortality differences	No	No	No	No	No	No	N/A
**DEC**							
Silhouette score							
Cluster partitioning	1: 300<br>2: 433	1: 386<br>2: 162<br>3: 195	1: 393<br>2: 105<br>3: 110<br>4: 135	1: 387<br>2: 106<br>3: 117<br>4: 47<br>5: 86	1: 68<br>2: 100<br>3: 46<br>4: 144<br>5: 290<br>6: 95	1: 100<br>2: 40<br>3: 88<br>4: 14<br>5: 24<br>6: 14<br>7: 18	1: 18<br>2: 42<br>3: 23<br>4: 76<br>5: 7<br>6: 13<br>7: 92<br>8: 2
In-ICU mortality differences	No	No	Yes*	Yes*	Yes*	Yes*	No
30-day mortality differences	No	No	Yes*	No	Yes*	Yes*	No
90-day mortality differences	No	No	Yes*	Yes*	Yes*	Yes*	No

K-means = k-means clustering, PCA = principal component analysis, HC = hierarchical clustering, HC-DTW = hierarchical clustering with dynamic time warping, DEC = Deep Embedded Clustering
$^{\#}$ Indicates $p < 0.05$, * Indicates $p < 0.01$
Due to the heavy computational requirements of HC-DTW, analysis was only performed for 2-7 clusters,

Figures S1-S8. Stability analysis of Deep Embedded Clustering partitioning for 4 to 7 clusters. Stability analysis uses the Jaccard coefficient, a cluster-wise similarity measure between sets which is calculated by the bootstrap distribution of the Jaccard coefficient for every single cluster of a clustering compared to the most similar cluster in the bootstrapped data sets. This can be applied to all cluster analysis methods, even not based on Euclidean data with a fixed number of clusters such as k-means. This way of assessing stability is therefore a reliable internal validity measure for Deep Embedded Clustering (DEC). The figures below show the Jaccard coefficients for 4 to 7 clusters for DEC. Stability for 2, 3, and 8 clusters was not reported as these partitions were not clinically relevant.

Figure S1. Entropy plots for DEC 4 clusters

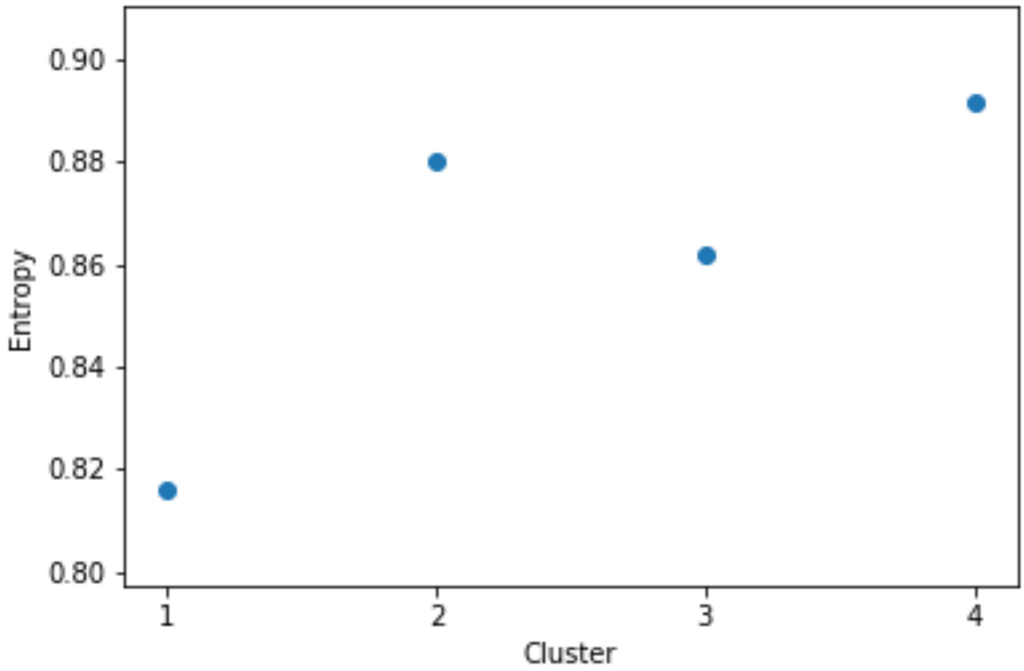

Figure S2. Jaccard score plots for DEC 4 clusters

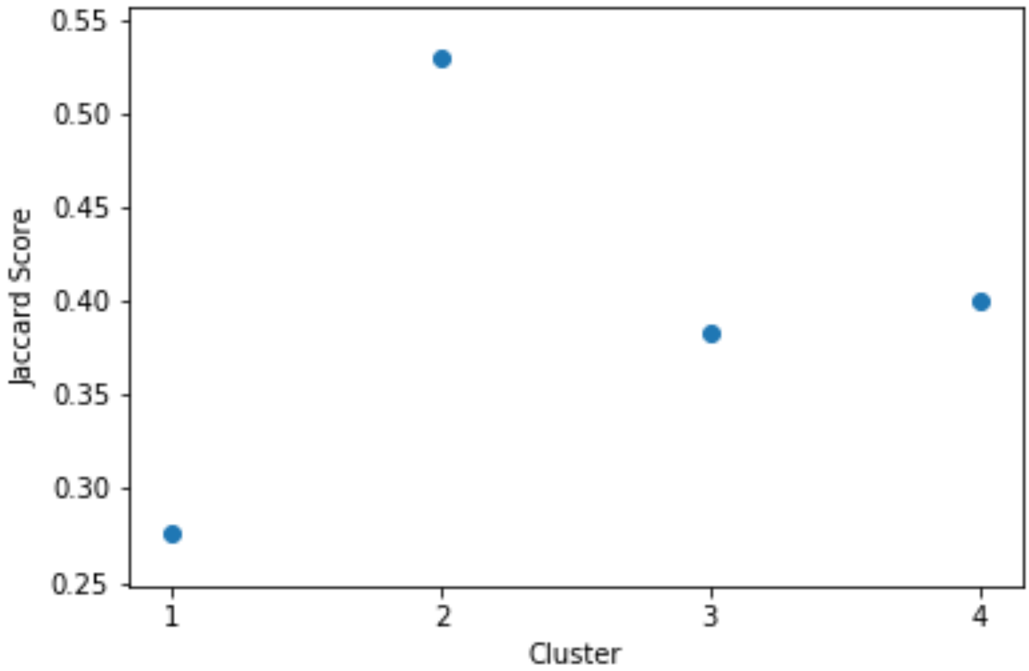

Figure S3. Entropy plot for DEC 5 clusters

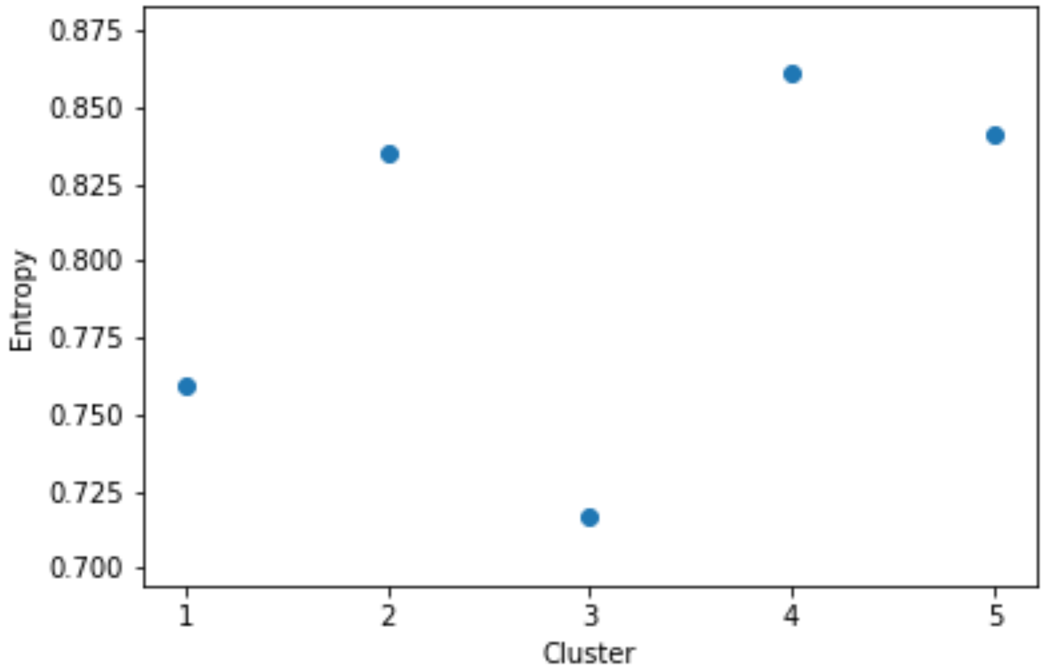

Figure S4. Jaccard score plot for DEC 5 clusters

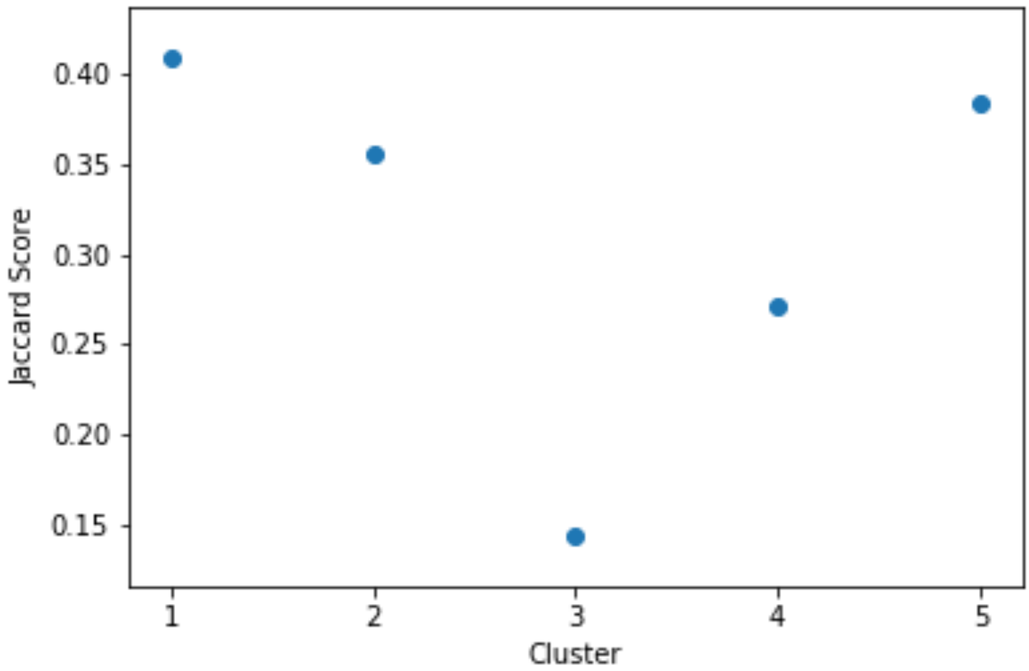

Figure S5. Entropy plot for DEC 6 clusters

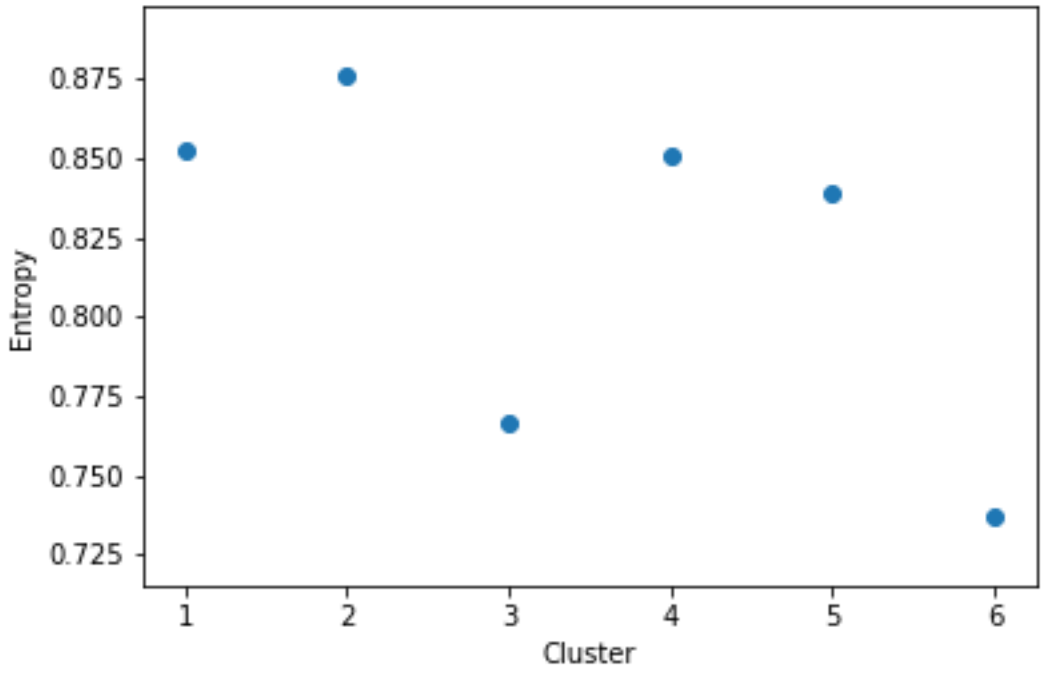

Figure S6. Jaccard score plot for DEC 6 clusters

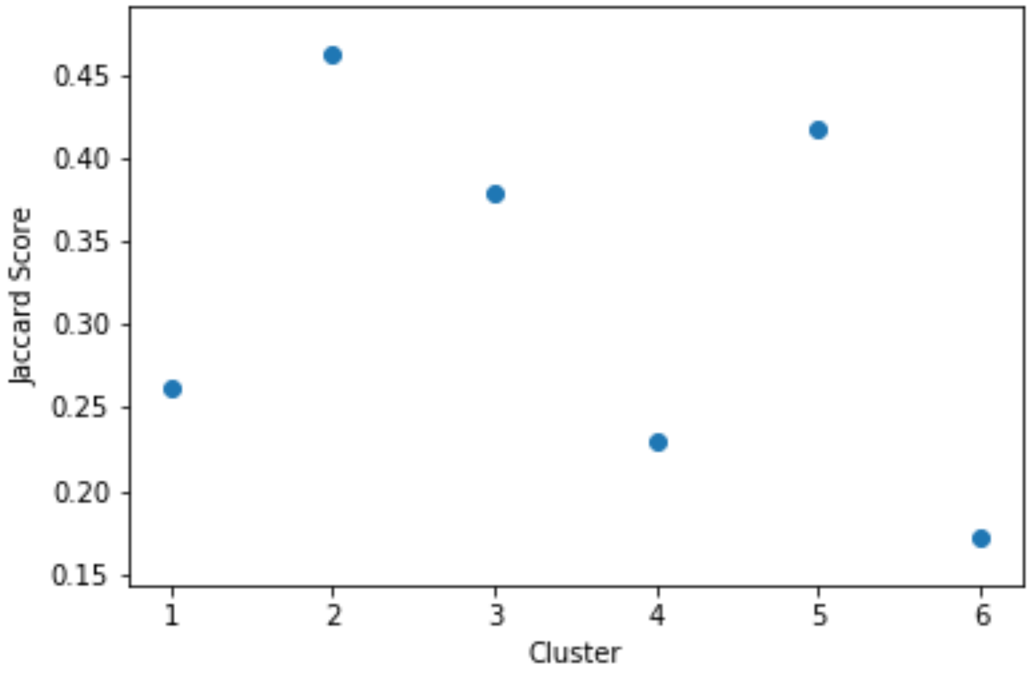

Figure S7. Entropy plot for DEC 7 clusters

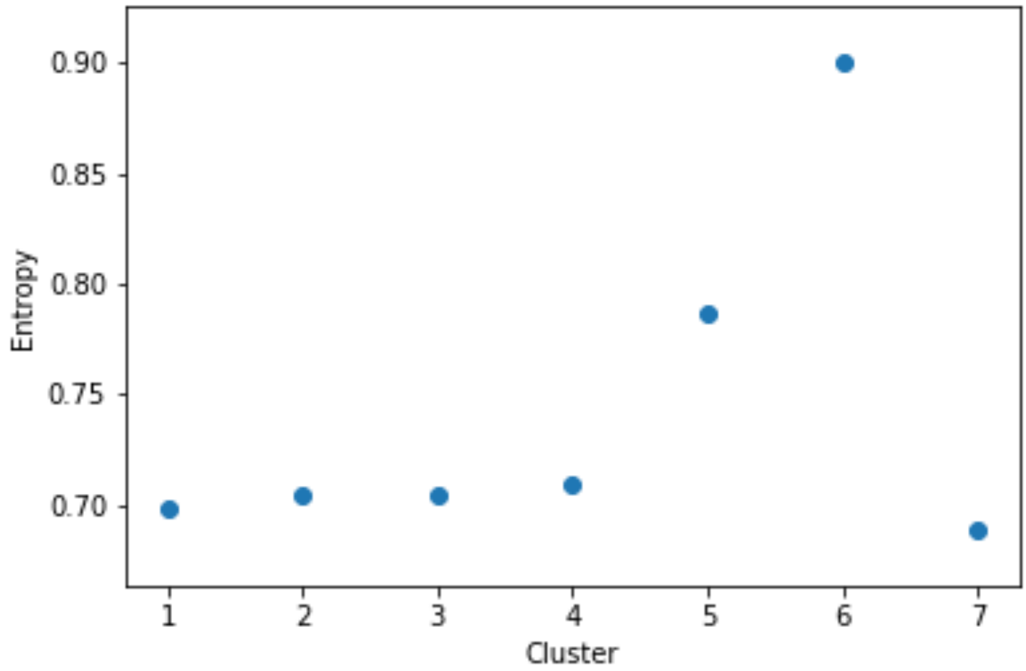

Figure S8. Jaccard score plots for DEC 7 clusters

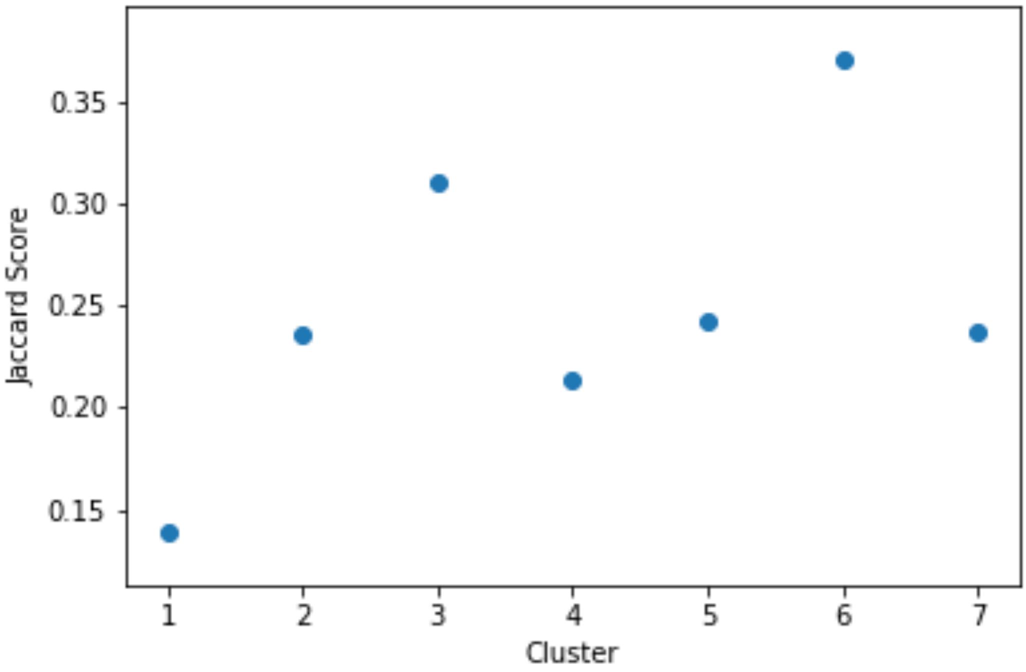

Table S2. Descriptive statistics of laboratory variables. The average number of measurements per variable per patient, and the average time between measurements for each variable across all patients, presented with the respective 95% confidence interval (CI).

Variable	Measurements per patient during stay	Time between consecutive measurements (in hours)
**ALAT**	4.8 [4.5,5.1]	14.1 [13.3,14.9]
**ASAT**	4.8 [4.5,5.1]	14.1 [13.3,14.9]
**Albumin**	4.2 [4.0,4.5]	14.7 [13.7,15.7]
**ALP**	4.5 [4.3,4.8]	14.4 [13.5,15.3]
**Amylase**	4.1 [3.8,4.3]	14.0 [13.1,15.0]
**Bilirubin (total)**	4.5 [4.2,4.7]	14.2 [13.4,15.1]
**CK**	7.1 [6.7,7.4]	12.1 [11.7,12.5]
**CRP**	6.4 [6.1,6.7]	13.8 [13.4,14.2]
**Calcium**	4.2 [4.0,4.5]	14.6 [13.6,15.6]
**Chloride**	5.8 [5.4,6.1]	13.9 [13.5,14.4]
**Total protein**	4.1 [3.8,4.3]	14.4 [13.4,15.4]
**Fibrinogen**	2.9 [2.6,3.2]	8.2 [7.0,9.3]
**Phosphate**	5.8 [5.4,6.1]	14.0 [13.5,14.4]
**Gamma-GT**	4.5 [4.3,4.8]	14.3 [13.4,15.3]
**Hb**	6.9 [6.6,7.3]	13.3 [12.9,13.6]
**Ht**	6.6 [6.3,7.0]	13.5 [13.1,13.9]
**Potassium**	1.2 [1.1,1.3]	3.6 [2.7,4.5]
**Creatinine**	6.6 [6.3,7.0]	13.6 [13.2,14.0]
**LDH**	6.3 [6.0,6.6]	13.6 [13.2,14.0]
**Leukocytes**	6.7 [6.4,7.1]	13.5 [13.1,13.8]
**MCV**	4.9 [4.6,5.2]	12.4 [11.7,13.0]
**Magnesium**	6.0 [5.6,6.3]	13.7 [13.3,14.2]
**Sodium**	6.6 [6.3,7.0]	13.5 [13.2,13.9]
**Ionized calcium**	35.2 [32.8,37.7]	3.1 [3.0,3.1]
**POC Glucose**	35.9 [33.4,38.3]	3.0 [3.0,3.1]
**POC Hb**	35.9 [33.4,38.3]	3.1 [3.0,3.2]
**POC HbCO**	26.2 [23.9,28.5]	3.5 [3.2,3.9]
**POC HbMet**	26.3 [24.0,28.6]	3.5 [3.2,3.9]
**POC Potassium**	35.9 [33.5,38.4]	3.1 [3.0,3.3]
**POC Lactate**	35.5 [33.1,37.9]	3.1 [3.0,3.2]
**POC Sodium**	35.8 [33.4,38.3]	3.0 [3.0,3.1]
**POC arterial HCO3**	34.5 [32.1,36.9]	3.2 [3.0,3.3]
**POC arterial pCO2**	34.7 [32.3,37.1]	3.1 [3.0,3.2]
**POC arterial pH**	34.7 [32.3,37.1]	3.1 [3.0,3.2]

**POC arterial pO2**	34.7 [32.3,37.1]	3.1 [3.0,3.2]
**POC arterial saturation**	34.9 [32.5,37.3]	3.1 [3.0,3.2]
**Thrombocytes**	7.7 [7.4,8.1]	12.1 [11.7,12.5]
**Troponin T**	5.5 [5.2,5.8]	12.4 [11.5,13.3]
**Urea**	6.6 [6.3,6.9]	13.6 [13.2,14.0]

ALAT = alanine transaminase, ASAT = aspartate transaminase, CK = creatine kinase, CRP = C-reactive protein, Hb = hemoglobin, Ht = hematocrit, LDH = lactate dehydrogenase, POC = point of care, HCO_3 = bicarbonate, pCO_2 = arterial CO_2 pressure, pO_2 = arterial O_2 pressure, HbMet = methemoglobin, HbCO = carboxyhemoglobin.

Table S3. Summary of the data from all non-laboratory variables in the six identified clusters. The p-values are from the ANOVA and Chi-square analysis for continuous and categorical binary variables between the six clusters. Clusters with significant differences determined by the pairwise t-test or Fisher's exact test for continuous and categorical binary variables. Bolded font shows relevant variables for each cluster.

**Variable**	**Cluster 1**	**Cluster 2**	**Cluster 3**	**Cluster 4**	**Cluster 5**	**Cluster 6**	**p-value**
**Patient characteristics**							
Age (years)	59.96	62.02	59.59	61.66	62.48	66.09	0.328
Gender (% female)	51.47	59.00	54.35	63.19	66.90	69.47	0.0859
ICU length of stay (days)	7.21	5.58	6.51	**2.80**	7.70	4.90	<0.001
APACHE IV Score	81.10	84.15	**100.45**	**61.94**	75.61	82.40	<0.001
SAPS II Score	47.41	52.18	**59.24**	**40.90**	46.00	46.48	<0.001
BMI	26.71	27.20	28.10	26.03	26.67	26.91	0.371
Surgical patient (%)	20.90	**48.00**	30.44	**38.19**	35.52	27.37	0.005
Previous admission to ICU (%)	10.29	**11.00**	8.70	11.11	12.76	11.58	0.967
**Admission diagnosis by organ system**							<0.001
Cardiovascular	22.39	55.00	**39.13**	28.47	32.07	22.11	
Gastrointestinal	**25.37**	12.00	26.09	4.86	13.10	**20.00**	
Genito-urinary	2.99	0.00	4.35	0.00	0.69	3.16	
Haematological	5.97	0.00	4.35	0.00	0.69	2.11	
Metabolic	4.48	2.00	2.17	4.17	0.69	3.16	
Musculoskeletal/skin	2.99	0.00	0.00	0.00	1.38	1.05	
Neurological	7.46	4.00	0.00	**38.89**	15.52	1.05	
Respiratory	**23.88**	9.00	6.52	9.03	22.41	**41.05**	
Transplant	4.48	14.00	15.22	0.00	3.10	5.26	
Trauma	0.00	4.00	2.17	**14.58**	10.35	1.05	
**Clinical examination**							
Mechanically ventilated at admission (%)	41.18	**81.00**	63.04	60.42	**67.59**	40.00	<0.001
Mottling (% with severe, >4)	4.41	3.00	10.87	**0.69**	2.41	4.21	0.0209
Atrial fibrillation (%)	7.35	3.00	6.52	4.17	7.93	12.63	0.0985
EMV score	12.96	11.21	11.02	**9.74**	10.88	13.74	<0.001
Respiratory rate (BPM)	**20.74**	17.86	18.17	16.31	17.67	**19.55**	<0.001
Heart rate at admission (bpm)	**106.78**	94.81	101.76	**82.60**	96.01	**99.44**	<0.001
Urine output in previous 6 hours (ml/kg/h)	0.86	0.77	**0.58**	**1.13**	0.93	0.75	<0.001
CRT prolonged (%)	26.47	35.00	**43.48**	**15.97**	**35.17**	25.26	<0.001
Worsened respiratory condition after 24 hours (%)	27.94	6.00	17.39	**4.86**	**14.14**	11.58	<0.001
Cardiac index > 2.2 (%)	48.53	**27.00**	45.65	**45.83**	**39.31**	**58.95**	<0.001
DBP	60.29	59.54	56.48	65.23	61.42	58.98	<0.001
SBP	117.78	110.73	107.29	129.30	118.61	118.77	<0.001
MAP	77.60	75.36	72.11	86.15	79.38	77.61	<0.001
CVP	8.50	8.36	9.06	7.50	9.66	9.76	0.796

**Co-morbidities and medical history**							
History of CVD (%)	2.94	9.00	2.17	**3.47**	5.52	4.21	0.330
History of CKD (%)	8.82	9.00	4.35	**0.69**	2.41	27.37	<0.001
History of cirrhosis (%)	**10.29**	5.00	15.22	**2.08**	0.69	1.05	<0.001
History of COPD (%)	8.82	10.00	4.35	**5.56**	**16.90**	**17.90**	0.0025
Previous dialysis (%)	1.47	2.00	2.17	**0.00**	0.00	6.32	<0.001
History of diabetes (%)	20.59	23.00	28.26	**11.81**	18.28	32.63	0.0025
History of hematological malignancy (%)	**16.18**	1.00	6.52	**1.39**	2.41	6.32	<0.001
History of metastatic disease (%)	7.35	3.00	8.70	**0.69**	3.10	5.26	0.058
History of myocardial infarction (%)	1.47	**21.00**	6.52	**7.64**	7.59	3.16	<0.001
History of respiratory insufficiency (%)	0.00	7.00	2.17	**2.78**	5.86	7.37	0.132

Table S4. Summary of the mean data from all laboratory variables in the six identified clusters. The p-values are from the ANOVA analysis for continuous variables between the six clusters. Data are presented as mean and 95% CI.

Laboratory variables	**Cluster 1**	**Cluster 2**	**Cluster 3**	**Cluster 4**	**Cluster 5**	**Cluster 6**	**p-value**
**ALAT (U/L)**	110.75 [66.15,155.35]	183.78 [147.07,220.49]	931.25 [629.68,1232.82]	32.0 [27.21,36.79]	55.01 [47.43,62.59]	31.08 [23.61,38.55]	<0.001
**ASAT (U/L)**	134.76 [91.71,177.82]	313.09 [236.83,389.36]	1470.27 [958.36,1982.19]	47.77 [38.64,56.9]	69.85 [62.1,77.6]	44.51 [35.78,53.24]	<0.001
**Albumin (g/L)**	25.62 [24.26,26.98]	27.99 [26.82,29.16]	25.75 [24.05,27.44]	35.07 [34.13,36.01]	29.76 [29.08,30.44]	27.86 [26.79,28.94]	<0.001
**Amylase (U/L)**	104.65 [66.32,142.98]	136.46 [108.42,164.5]	203.77 [116.36,291.19]	56.24 [51.12,61.37]	78.78 [70.88,86.67]	54.76 [45.64,63.87]	<0.001
**ALP (U/L)**	195.02 [163.69,226.35]	88.39 [75.95,100.83]	270.39 [101.86,438.92]	62.65 [59.29,66.02]	83.47 [77.94,89.0]	85.55 [77.23,93.86]	<0.001
**Bilirubin total (µmol/L)**	37.53 [24.15,50.91]	13.42 [11.68,15.15]	86.21 [46.95,125.46]	9.89 [8.84,10.94]	9.44 [8.78,10.1]	9.77 [8.63,10.9]	<0.001
**Gamma-GT (U/L)**	249.09 [196.16,302.02]	93.67 [69.51,117.84]	236.26 [139.97,332.56]	37.84 [32.0,43.69]	75.25 [66.99,83.52]	59.43 [50.44,68.41]	<0.001
**CK (U/L)**	179.53 [126.13,232.93]	1965.37 [770.91,3159.82]	4105.44 [739.69,7471.2]	807.95 [270.05,1345.86]	556.52 [462.8,650.24]	262.38 [167.38,357.38]	<0.001
**CRP (mg/L)**	142.4 [116.13,168.67]	71.45 [59.43,83.47]	77.49 [60.63,94.36]	40.87 [33.31,48.42]	94.99 [86.65,103.34]	175.71 [153.28,198.14]	<0.001
**Calcium (mmol/L)**	2.0 [1.96,2.04]	2.01 [1.98,2.05]	1.99 [1.94,2.04]	2.12 [2.09,2.14]	2.04 [2.03,2.06]	2.05 [2.01,2.08]	0.0035
**Chloride (mEq/L)**	103.32 [101.3,105.34]	104.86 [103.82,105.89]	103.79 [102.03,105.54]	103.84 [103.08,104.61]	104.07 [103.52,104.63]	102.18 [101.16,103.21]	<0.001
**Magnesium (mmol/L)**	0.84 [0.79,0.89]	0.92 [0.88,0.96]	0.89 [0.84,0.94]	0.79 [0.77,0.8]	0.84 [0.82,0.85]	0.86 [0.82,0.9]	0.043
**MCV (µL)**	93.01 [91.02,95.0]	89.69 [88.42,90.97]	91.95 [89.79,94.11]	88.92 [87.98,89.87]	90.74 [90.04,91.44]	90.06 [88.65,91.46]	<0.001
**Sodium (mmol/L)**	139.09 [137.22,140.95]	139.71 [138.85,140.58]	139.31 [137.95,140.67]	138.93 [138.23,139.63]	139.57 [139.12,140.01]	137.97 [137.05,138.89]	<0.001
**Phosphate (mmol/L)**	1.11 [1.03,1.2]	1.15 [1.07,1.23]	1.38 [1.25,1.5]	0.91 [0.88,0.94]	1.02 [0.99,1.04]	1.33 [1.23,1.44]	<0.001
**Potassium (mmol/L)**	3.94 [3.79,4.1]	4.26 [3.98,4.53]	4.49 [4.16,4.82]	3.87 [3.78,3.96]	4.27 [4.19,4.36]	4.5 [4.22,4.78]	<0.001
**Fibrinogen (g/L)**	4.28 [3.74,4.81]	2.65 [2.39,2.91]	2.67 [2.22,3.12]	2.73 [2.57,2.9]	3.58 [3.38,3.77]	5.33 [4.88,5.78]	<0.001
**Hb (mmol/L)**	5.9 [5.62,6.18]	6.68 [6.42,6.95]	5.97 [5.63,6.3]	7.51 [7.32,7.7]	6.84 [6.69,6.98]	6.18 [5.93,6.42]	<0.001
**Ht**	0.29 [0.28,0.3]	0.33 [0.32,0.34]	0.29 [0.27,0.31]	0.36 [0.35,0.37]	0.34 [0.33,0.35]	0.31 [0.3,0.32]	<0.001
**Creatinine (µmol/L)**	118.64 [100.14,137.15]	118.53 [102.85,134.21]	154.62 [125.12,184.12]	72.58 [68.69,76.48]	82.19 [78.08,86.3]	213.47 [173.7,253.24]	<0.001
**LDH (U/L)**	427.62 [356.71,498.52]	614.24 [530.08,698.4]	1579.34 [1173.84,1984.84]	230.54 [216.55,244.53]	306.44 [287.94,324.93]	284.09 [252.54,315.64]	<0.001
**Leukocytes (x10^9/L)**	12.39 [10.14,14.64]	16.7 [14.82,18.58]	23.03 [15.37,30.7]	11.65 [11.13,12.16]	13.81 [13.29,14.33]	12.65 [11.22,14.07]	<0.001
**Thrombocytes (x10^9/L)**	183.19 [150.09,216.29]	193.2 [174.89,211.51]	175.87 [140.99,210.74]	207.61 [196.96,218.26]	256.99 [244.37,269.61]	240.4 [213.95,266.85]	<0.001
**Troponin T (ng/L)**	114.7 [28.51,200.9]	1710.57 [1169.53,2251.61]	2023.1 [578.47,3467.73]	131.96 [87.35,176.57]	278.87 [196.7,361.04]	263.73 [86.79,440.68]	<0.001
**Total protein (g/L)**	52.65 [50.73,54.57]	49.63 [47.96,51.3]	47.98 [45.48,50.48]	58.26 [56.75,59.78]	54.74 [53.77,55.7]	54.69 [53.0,56.38]	<0.001
**Urea (mmol/L)**	11.19 [9.26,13.12]	10.38 [9.08,11.69]	13.41 [11.25,15.56]	5.79 [5.45,6.12]	8.07 [7.63,8.52]	15.61 [13.7,17.53]	<0.001
**Ionized calcium (mmol/L)**	1.12 [1.11,1.14]	1.13 [1.12,1.15]	1.12 [1.09,1.14]	1.16 [1.15,1.17]	1.14 [1.14,1.15]	1.13 [1.11,1.15]	<0.001
**POC Glucose (mmol/L)**	7.98 [7.53,8.44]	8.82 [8.5,9.15]	8.86 [8.04,9.69]	7.74 [7.54,7.94]	7.93 [7.79,8.06]	7.91 [7.59,8.23]	<0.001
**POC Hb (mmol/L)**	5.94 [5.66,6.22]	6.68 [6.41,6.94]	6.02 [5.67,6.37]	7.55 [7.36,7.75]	6.85 [6.7,6.99]	6.24 [6.0,6.48]	0.0109

**POC Potassium (mmol/L)**	3.98 [3.88,4.08]	4.25 [4.16,4.34]	4.36 [4.21,4.52]	3.93 [3.88,3.98]	4.12 [4.09,4.16]	4.28 [4.18,4.38]	<0.001
**POC Lactate (mmol/L)**	1.6 [1.35,1.84]	2.51 [2.28,2.74]	3.77 [2.97,4.57]	1.38 [1.26,1.49]	1.54 [1.44,1.63]	1.39 [1.25,1.53]	<0.001
**POC Sodium (mmol/L)**	137.84 [136.0,139.67]	137.53 [136.63,138.42]	137.12 [136.09,138.15]	137.95 [137.25,138.65]	138.01 [137.53,138.48]	136.52 [135.57,137.47]	<0.001
**POC art HCO3 (mmol/L)**	23.31 [22.2,24.41]	22.09 [21.27,22.92]	20.0 [18.89,21.11]	23.64 [23.25,24.03]	24.47 [23.98,24.95]	22.26 [21.16,23.35]	<0.001
**POC art pCO2 (kPa)**	5.14 [4.9,5.38]	5.12 [4.96,5.28]	4.73 [4.51,4.95]	5.03 [4.93,5.13]	5.41 [5.29,5.53]	5.13 [4.88,5.37]	<0.001
**POC art pH**	7.4 [7.38,7.41]	7.37 [7.36,7.38]	7.36 [7.34,7.38]	7.41 [7.41,7.42]	7.4 [7.39,7.4]	7.38 [7.36,7.39]	<0.001
**POC art pO2 (kPa)**	11.83 [11.32,12.34]	15.08 [14.31,15.85]	13.85 [12.65,15.06]	14.31 [13.76,14.86]	12.07 [11.82,12.32]	11.56 [11.08,12.05]	<0.001
**POC art saturation (%)**	96.18 [95.78,96.59]	96.28 [95.82,96.73]	96.04 [95.3,96.78]	97.26 [96.99,97.52]	95.89 [95.63,96.14]	95.02 [94.22,95.81]	<0.001
**POC HbMet**	0.92 [0.78,1.06]	1.36 [1.26,1.45]	0.98 [0.83,1.13]	0.96 [0.89,1.04]	0.99 [0.93,1.05]	1.03 [0.91,1.14]	<0.001
**POC HbCO**	1.54 [1.38,1.7]	1.21 [1.12,1.3]	1.51 [1.34,1.67]	1.33 [1.22,1.44]	1.29 [1.21,1.36]	1.55 [1.4,1.71]	<0.001

ALAT = alanine transaminase, ASAT = aspartate transaminase, CK = creatine kinase, CRP = C-reactive protein, Hb = hemoglobin, Ht = hematocrit, LDH = lactate dehydrogenase, POC = point of care, HCO_3 = bicarbonate, pCO_2 = arterial CO_2 pressure, pO_2 = arterial O_2 pressure, HbMet = methemoglobin, HbCO = carboxyhemoglobin.

Figure S9. Heatmap of mean values for laboratory variables per cluster

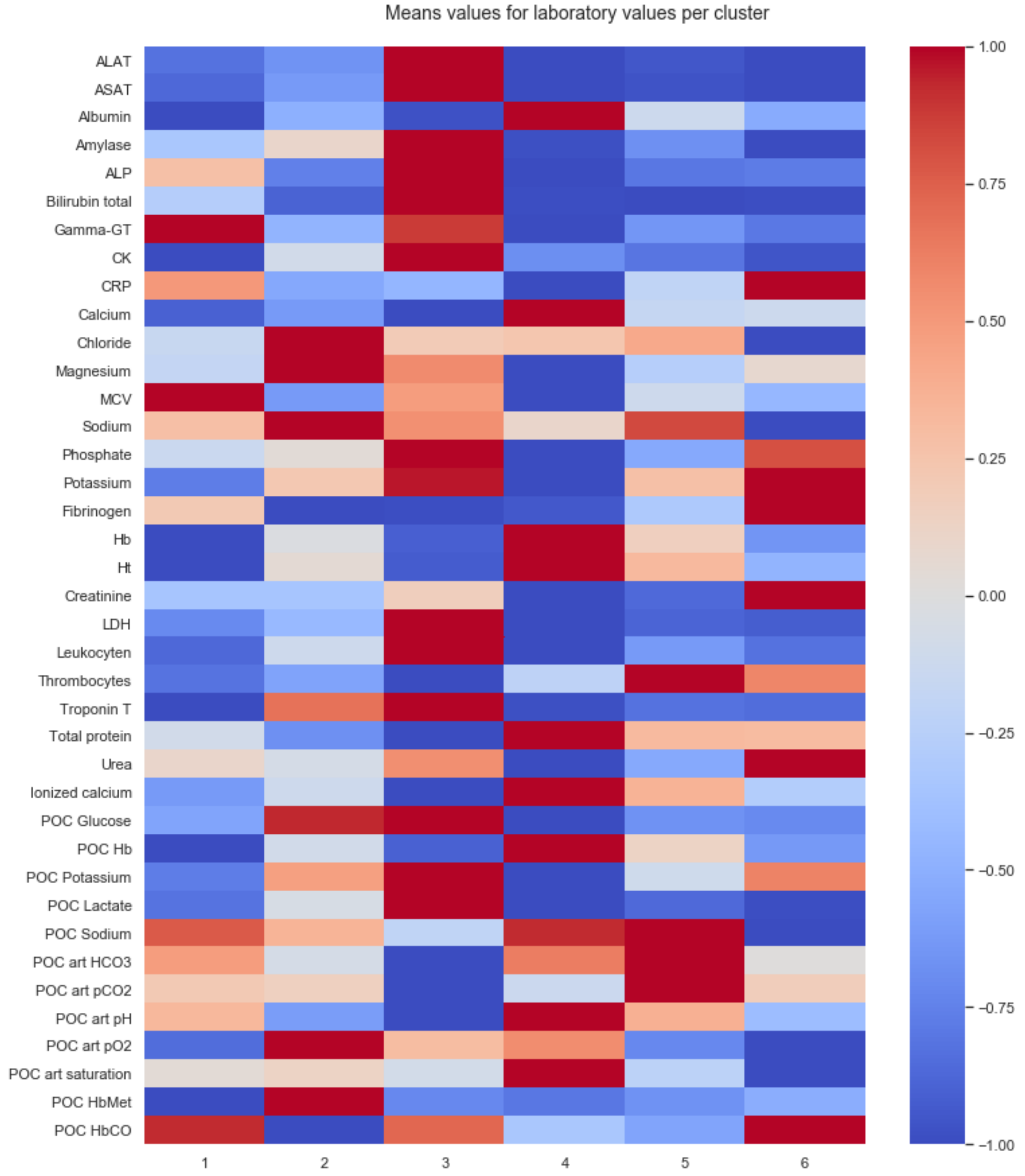

Table S5. Summary of the variance data from all laboratory variables in the six identified clusters. The p-values are from the ANOVA analysis for continuous variables between the six clusters. Data are presented as mean and 95% CI.

Lab variables	Cluster 1	Cluster 2	Cluster 3	Cluster 4	Cluster 5	Cluster 6	p-value
**ALAT**	28.56 [13.56,43.57]	102.12 [73.06,131.17]	611.1 [341.53,880.66]	4.93 [3.54,6.31]	16.68 [13.98,19.39]	5.67 [3.67,7.67]	<0.001
**ASAT**	52.49 [30.43,74.55]	203.81 [143.76,263.86]	1260.02 [708.64,1811.4]	11.13 [5.66,16.6]	26.85 [22.28,31.42]	10.59 [6.93,14.26]	<0.001
**Albumin**	1.98 [1.71,2.24]	3.08 [2.6,3.57]	2.76 [2.3,3.21]	1.1 [0.88,1.32]	2.44 [2.23,2.65]	1.84 [1.5,2.19]	<0.001
**Alkaline phosphatase**	55.88 [18.48,93.27]	64.23 [44.9,83.55]	94.4 [47.87,140.93]	5.04 [3.82,6.27]	30.09 [23.75,36.44]	9.61 [6.64,12.57]	<0.001
**Bilirubin (total)**	34.47 [23.51,45.44]	20.04 [15.56,24.53]	80.76 [45.8,115.72]	3.76 [3.07,4.46]	15.04 [13.09,16.99]	9.57 [6.9,12.24]	<0.001
**Gamma-GT**	6.57 [4.82,8.31]	4.37 [3.11,5.63]	26.79 [13.26,40.32]	1.35 [1.02,1.68]	2.65 [2.32,2.98]	1.78 [1.33,2.24]	<0.001
**CK**	52.03 [36.37,67.7]	22.0 [16.17,27.82]	77.53 [40.61,114.45]	3.85 [2.7,5.0]	21.78 [18.24,25.33]	10.21 [6.76,13.65]	<0.001
**CRP**	84.53 [53.65,115.41]	886.87 [504.33,1269.41]	2800.5 [695.29,4905.71]	266.33 [110.5,422.17]	325.26 [260.8,389.73]	120.33 [60.66,180.01]	<0.001
**Calcium**	45.74 [36.44,55.05]	45.59 [38.35,52.84]	40.21 [31.96,48.45]	27.4 [22.91,31.88]	53.26 [49.09,57.42]	45.78 [37.99,53.58]	<0.001
**Chloride**	0.08 [0.07,0.1]	0.1 [0.09,0.12]	0.11 [0.09,0.12]	0.04 [0.03,0.04]	0.07 [0.07,0.08]	0.08 [0.06,0.1]	<0.001
**Magnesium**	3.23 [2.57,3.88]	2.8 [2.45,3.15]	3.43 [2.76,4.11]	1.9 [1.66,2.13]	2.57 [2.42,2.73]	2.01 [1.73,2.29]	<0.001
**Sodium**	0.1 [0.08,0.11]	0.17 [0.15,0.19]	0.13 [0.11,0.16]	0.07 [0.06,0.08]	0.11 [0.1,0.11]	0.11 [0.09,0.13]	<0.001
**Phosphate**	1.28 [1.01,1.56]	1.71 [1.36,2.06]	2.4 [1.75,3.04]	0.96 [0.85,1.07]	1.45 [1.32,1.57]	1.09 [0.93,1.24]	<0.001
**Potassium**	2.96 [2.35,3.56]	2.43 [2.1,2.75]	2.95 [2.48,3.42]	1.75 [1.57,1.93]	2.49 [2.33,2.65]	2.03 [1.8,2.26]	0.149
**Fibrinogen**	0.25 [0.2,0.29]	0.28 [0.24,0.32]	0.42 [0.34,0.49]	0.16 [0.14,0.17]	0.23 [0.22,0.25]	0.23 [0.19,0.27]	<0.001
**Hb**	0.09 [0.04,0.14]	0.1 [0.05,0.16]	0.09 [0.03,0.15]	0.03 [0.02,0.05]	0.07 [0.04,0.1]	0.09 [0.03,0.15]	<0.001
**Ht**	0.29 [0.18,0.41]	0.53 [0.43,0.64]	0.53 [0.36,0.7]	0.19 [0.12,0.25]	0.47 [0.39,0.55]	0.13 [0.07,0.18]	<0.001
**Creatinine**	0.5 [0.43,0.58]	0.82 [0.73,0.9]	0.71 [0.61,0.81]	0.5 [0.46,0.55]	0.76 [0.71,0.8]	0.44 [0.39,0.5]	<0.001
**LDH**	0.02 [0.02,0.03]	0.04 [0.04,0.04]	0.04 [0.03,0.04]	0.02 [0.02,0.03]	0.04 [0.03,0.04]	0.02 [0.02,0.02]	<0.001
**Leukocytes**	23.3 [17.61,28.99]	25.6 [19.93,31.28]	41.39 [30.28,52.5]	6.59 [5.82,7.36]	14.01 [11.55,16.48]	38.81 [20.18,57.43]	<0.001
**Thrombocytes**	89.24 [62.28,116.19]	247.56 [190.44,304.69]	1101.51 [738.47,1464.55]	33.55 [27.42,39.67]	66.26 [55.25,77.28]	56.72 [36.16,77.29]	<0.001
**Troponin T**	2.68 [2.12,3.24]	4.28 [3.73,4.83]	8.07 [4.27,11.88]	2.07 [1.89,2.26]	3.39 [3.16,3.62]	2.52 [2.11,2.93]	<0.001
**Urea**	39.91 [29.12,50.7]	42.77 [37.65,47.89]	52.6 [30.68,74.53]	23.08 [20.75,25.4]	57.05 [51.26,62.83]	28.98 [24.74,33.22]	<0.001
**POC Calcium (ionized)**	47.56 [-8.64,103.76]	868.59 [604.76,1132.41]	1279.25 [401.59,2156.92]	54.8 [30.51,79.08]	137.08 [93.81,180.35]	64.87 [24.4,105.34]	<0.001
**POC Glucose**	2.48 [2.09,2.86]	5.57 [4.61,6.53]	5.1 [3.64,6.55]	1.46 [1.16,1.76]	3.78 [3.44,4.12]	2.16 [1.75,2.57]	<0.001
**POC Hb**	2.21 [1.7,2.72]	2.78 [2.11,3.44]	3.79 [2.84,4.74]	0.82 [0.72,0.92]	1.72 [1.53,1.9]	2.24 [1.79,2.7]	<0.001

**POC Potassium**	0.04 [0.03,0.04]	0.05 [0.05,0.06]	0.06 [0.05,0.07]	0.03 [0.02,0.03]	0.04 [0.04,0.04]	0.04 [0.03,0.05]	<0.001
**POC Lactate**	1.96 [1.61,2.31]	2.79 [2.53,3.06]	2.89 [2.11,3.66]	1.36 [1.2,1.52]	1.76 [1.65,1.87]	1.77 [1.55,2.0]	<0.001
**POC Sodium**	0.43 [0.37,0.48]	0.75 [0.69,0.82]	0.65 [0.56,0.74]	0.42 [0.38,0.45]	0.62 [0.59,0.65]	0.41 [0.37,0.46]	<0.001
**POC art HCO_3**	0.37 [0.31,0.43]	0.48 [0.44,0.52]	0.56 [0.45,0.68]	0.3 [0.28,0.33]	0.4 [0.37,0.43]	0.35 [0.31,0.38]	<0.001
**POC art pCO_2**	0.57 [0.41,0.73]	1.69 [1.47,1.92]	2.16 [1.69,2.63]	0.56 [0.48,0.65]	0.72 [0.66,0.79]	0.53 [0.44,0.62]	<0.001
**POC art pH**	2.65 [2.09,3.21]	2.32 [2.04,2.6]	2.67 [2.27,3.08]	1.65 [1.51,1.78]	2.33 [2.18,2.49]	1.75 [1.57,1.94]	<0.001
**POC art pO_2**	2.0 [1.62,2.37]	2.59 [2.35,2.82]	3.15 [2.74,3.55]	1.34 [1.23,1.45]	2.32 [2.18,2.46]	1.81 [1.58,2.04]	<0.001
**POC art saturation**	0.67 [0.55,0.78]	0.73 [0.66,0.8]	0.7 [0.59,0.8]	0.45 [0.42,0.49]	0.75 [0.7,0.81]	0.53 [0.47,0.59]	<0.001
**POC HbMet**	0.19 [0.15,0.23]	0.27 [0.24,0.3]	0.29 [0.24,0.33]	0.18 [0.15,0.2]	0.19 [0.18,0.21]	0.17 [0.15,0.2]	<0.001
POC HbCO	0.21 [0.17,0.24]	0.3 [0.27,0.33]	0.35 [0.29,0.41]	0.26 [0.22,0.31]	0.27 [0.24,0.3]	0.21 [0.18,0.25]	0.0023

ALAT = alanine transaminase, ASAT = aspartate transaminase, Gamma-GT = Gamma glutamyltransferase, CK = creatine kinase, CRP = C-reactive protein, Hb = hemoglobin, Ht = hematocrit, LDH = lactate dehydrogenase, POC = point of care, HCO_3 = bicarbonate, pCO_2 = arterial CO_2 pressure, pO_2 = arterial O_2 pressure, HbMet = Methemoglobin, HbCO = Carboxyhemoglobin.

Figure S10. Heatmap with variance for laboratory variables

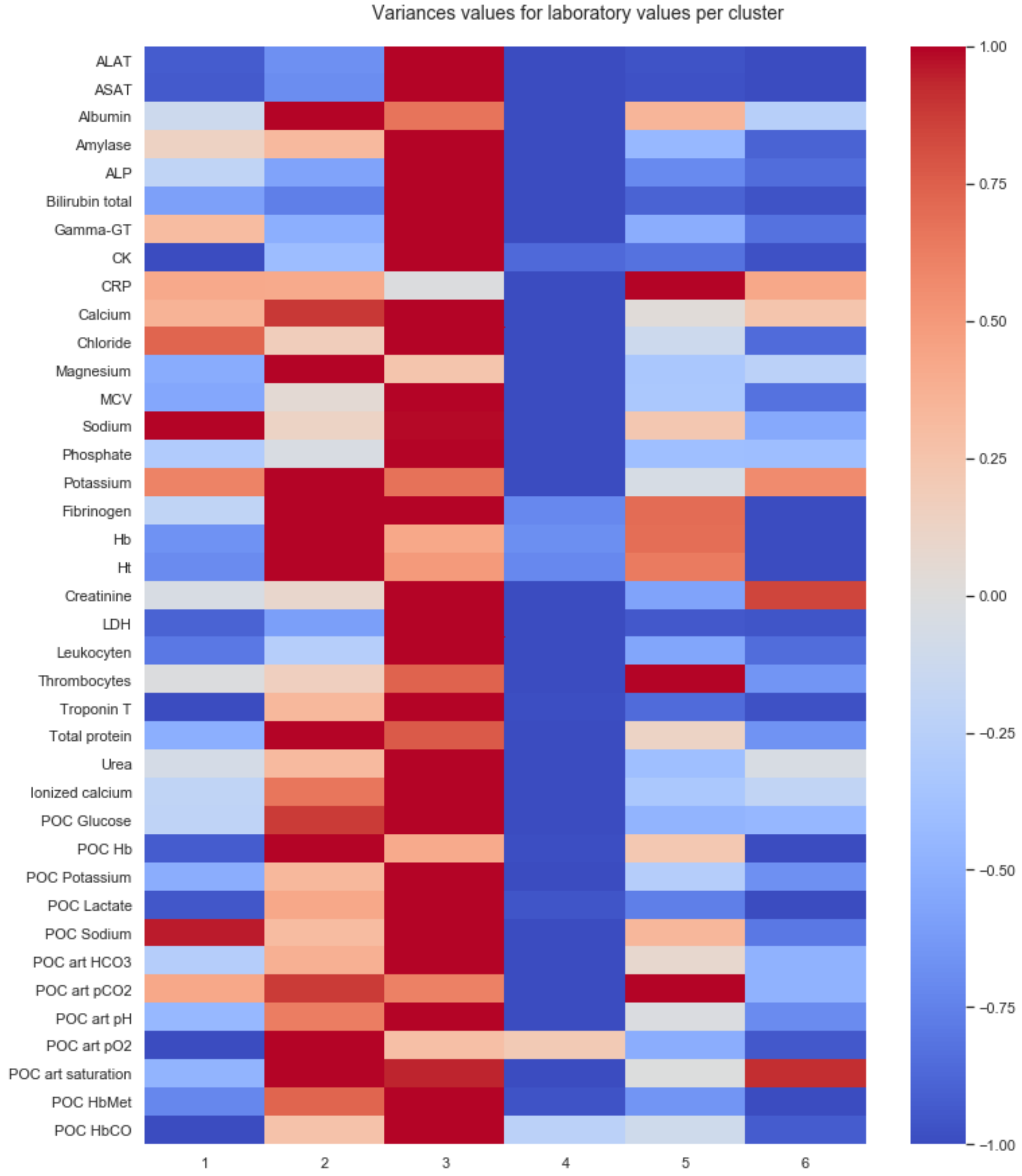

Table S6. Primary discharge diagnosis and outcomes per cluster.

Primary discharge diagnosis category	Cluster 1	Cluster 2	Cluster 3	Cluster 4	Cluster 5	Cluster 6	p-value
Cardiac	1.5	0	0	1.4	3.4	1.1	0.2026
Cardiac arrest	2.9	**30**	**10.9**	10.4	**12.4**	3.2	<0.001
Electrolyte disturbances	5.9	2	0	1.4	0.3	2.1	0.0259
Gastro	1.5	0	2.2	0	1.4	0	0.3702
Hemodynamic instability/non-cardiac shock	1.5	8	8.7	6.9	3.8	5.3	0.2487
Infection	7.4	1	2.2	0.7	1.4	2.1	0.0236
Liver	1.5	0	**8.7**	0	0.7	1.1	<0.001
Metabolic	0	1	4.3	0.7	0	0	0.0088
Neuro	2.9	1	0	**36.1**	**10.7**	0	<0.001
Other	1.5	0	0	1.4	0	2.1	0.1783
Post transplant - liver	4.4	7	**15.2**	0	0	0	<0.001
Post transplant - other	0	8	0	0	3.8	3.2	0.0037
Postoperative - cardiac	0	**9**	0	2.1	4.5	1.1	0.0056
Postoperative care - gastro	1.5	3	8.7	1.4	3.8	5.3	0.1906
Postoperative care - neuro	1.5	0	0	3.5	1.4	0	0.1454
Postoperative care - other	1.5	0	2.2	1.4	0.7	1.1	0.7883
Postoperative care - vascular	1.5	6	6.5	2.8	2.1	1.1	0.1505
Renal	2.9	0	0	0	0	2.1	0.0111
Respiratory failure	**25**	7	8.7	6.9	**19.3**	**18.9**	<0.001
Respiratory infection	**8.8**	2	0	0.7	6.9	**9.5**	0.0031
Sepsis	**20.6**	7	**10.9**	2.8	7.6	**31.6**	<0.001
Trauma	4.4	3	0	**16.7**	**12.4**	2.1	<0.001
**Outcome**							
Length of stay (days)	**7.2**	5.6	6.5	**2.8**	**7.7**	4.9	<0.001
In-ICU mortality	19.1	**30**	**34.8**	11.1	17.9	16.8	<0.001
30-day mortality	19.1	**31**	**37**	16.7	22.4	17.9	0.0275
90-day mortality	26.5	**36**	**43.5**	18.1	27.6	26.3	0.0067
No AKI during ICU stay	30.9	27	**8.7**	**69.4**	43.4	22.1	0.0085
AKI 1	19.1	17	17.4	11.1	11.4	29.5	<0.001
AKI 2 or AKI 3	50	56	**73.9**	**19.4**	45.2	48.4	<0.001
Required vasoactive medication	45.6	73	**80.4**	31.2	47.2	49.5	<0.001
Required RRT	14.7	14	**34.8**	1.4	2.8	13.7	<0.001

AKI = acute kidney injury, RRT = renal replacement therapy.

Table S7. Accuracy, sensitivity and specificity for XGBoost predicting cluster membership per cluster.

Cluster	Accuracy	Sensitivity/Recall	Specificity
1	75.0	64.3	97.8
2	78.3	90.0	96.1
3	77.8	77.8	98.5
4	95.8	79.3	99.2
5	78.8	89.7	84.6
6	100.0	78.9	100.0

Figure S11. Confusion matrix for XGBoost test set

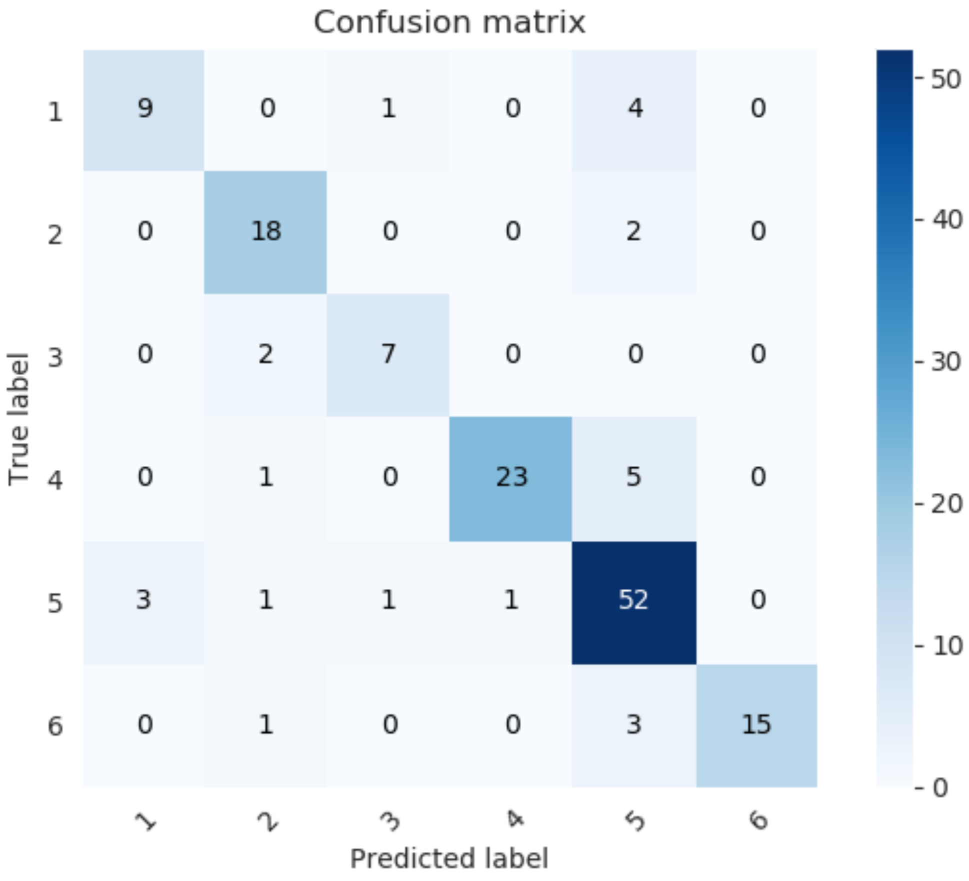

Figure S12. Top 10 variables per SHAP value for cluster 1

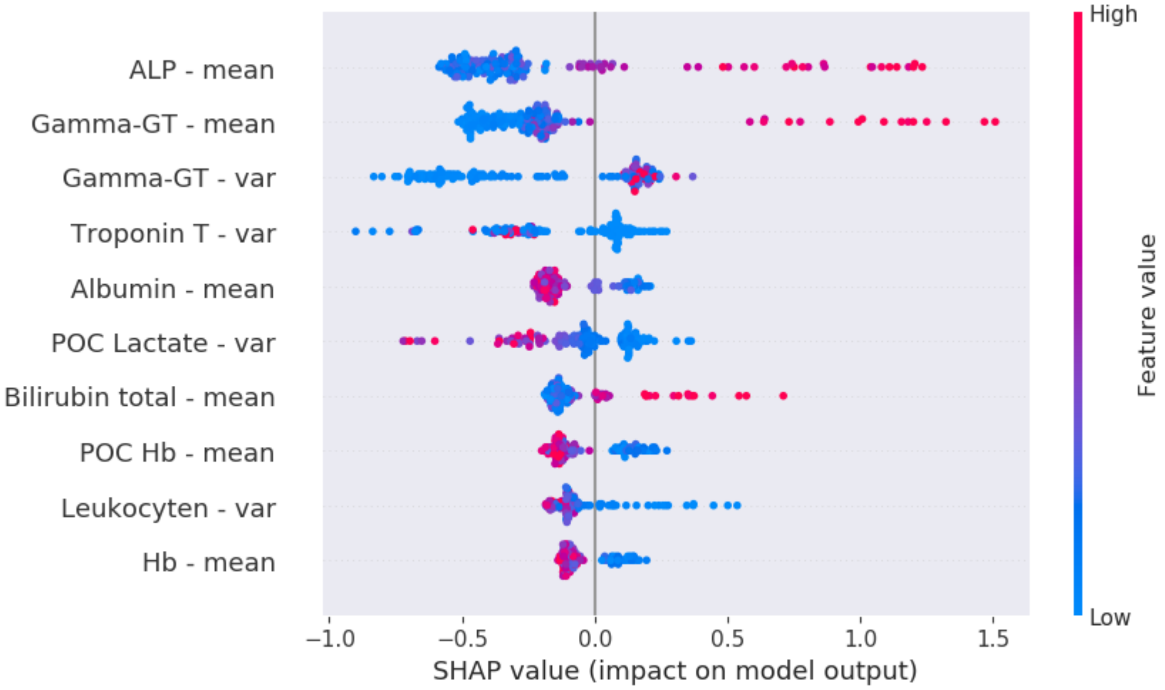

Figure S13. Top 10 variables per SHAP value for cluster 2

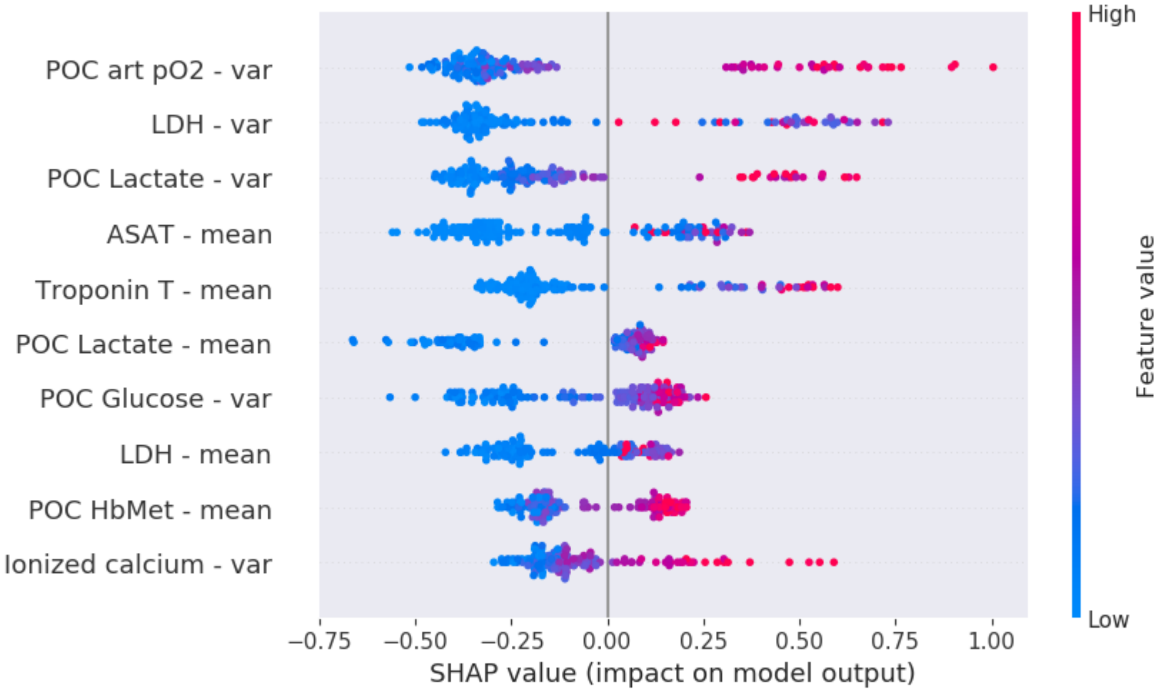

Figure S14. Top 10 variables per SHAP value for cluster 3

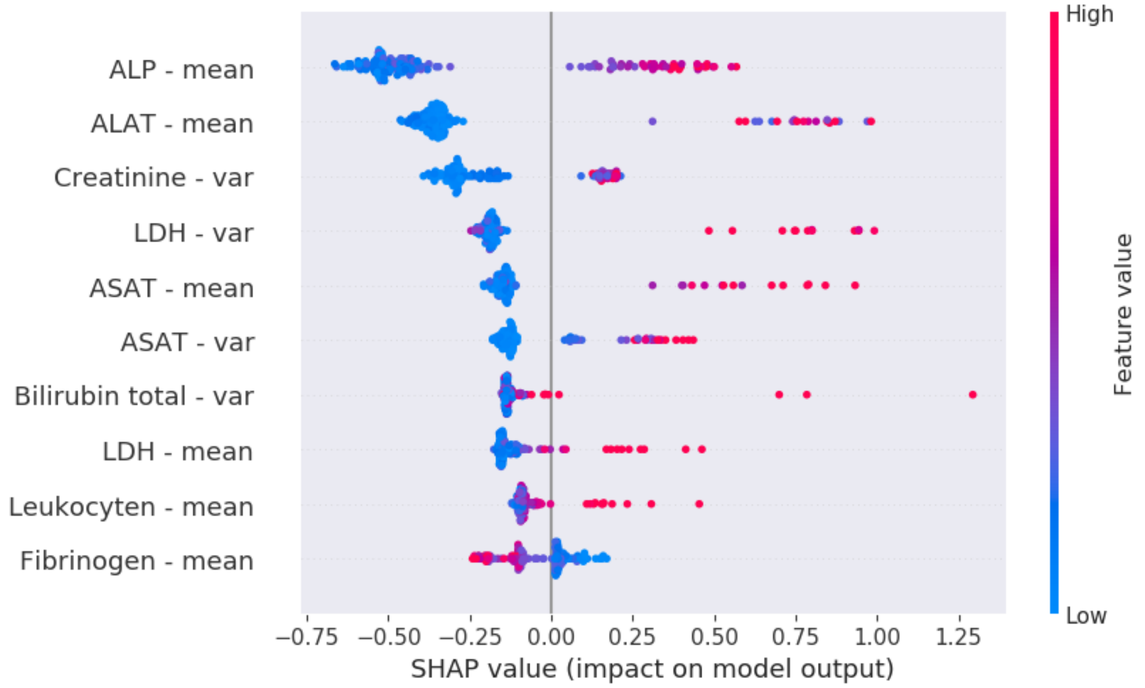

Figure S15. Top 10 variables per SHAP value for cluster 4

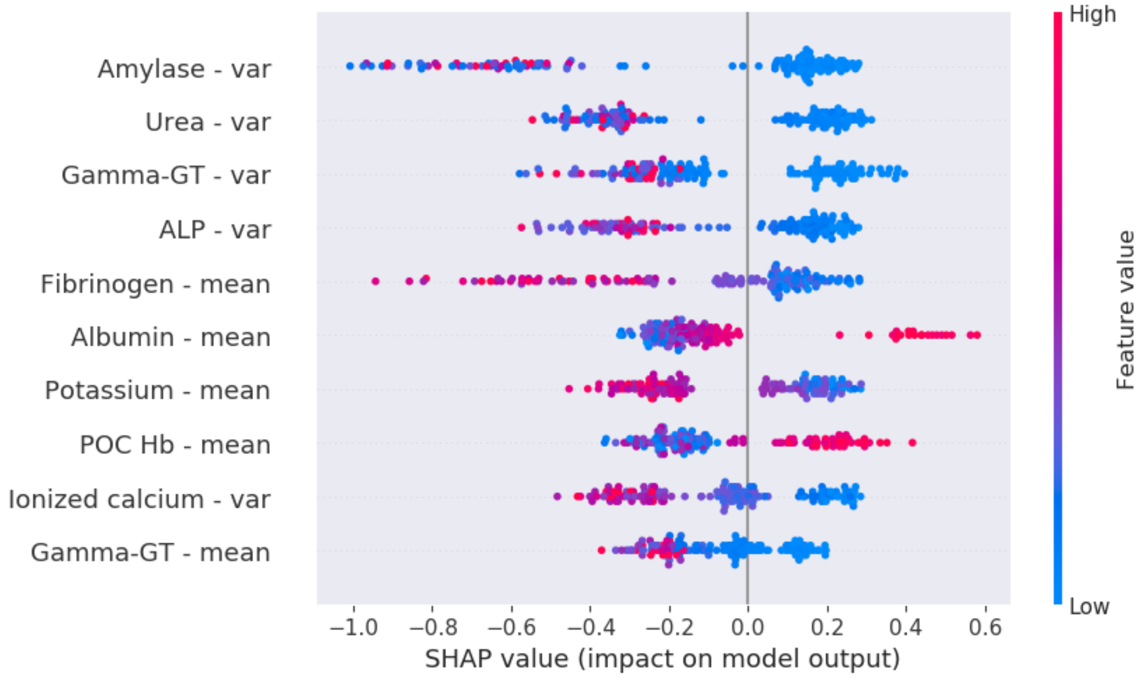

Figure S16. Top 10 variables per SHAP value for cluster 5

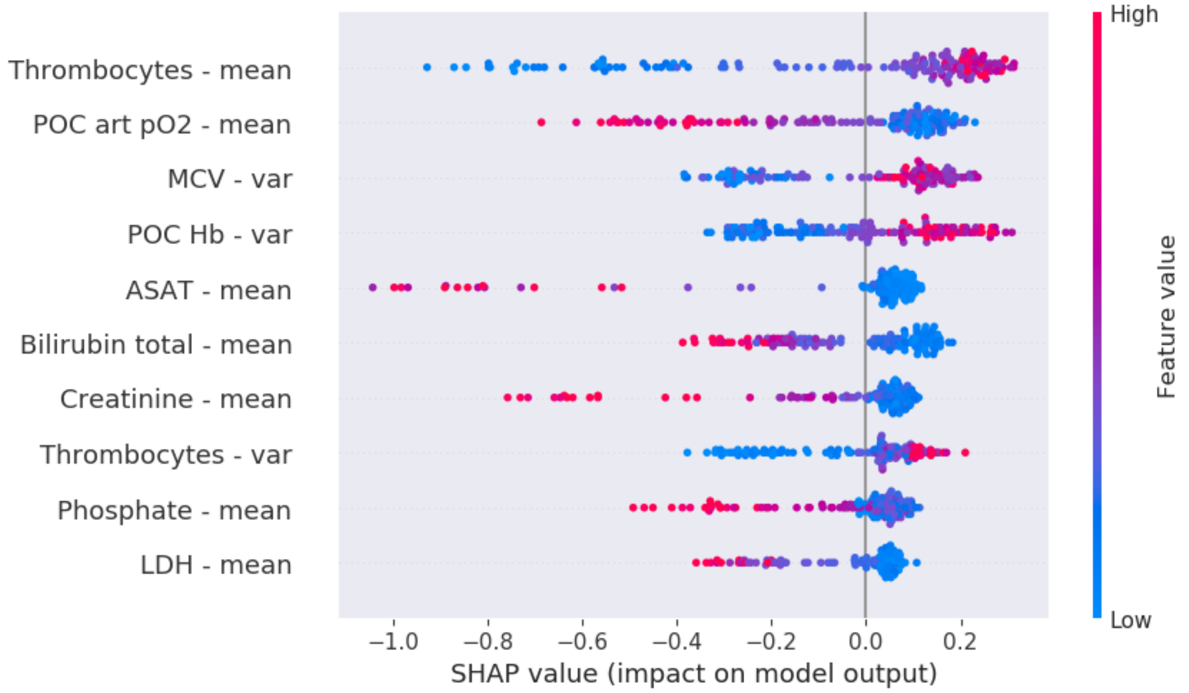

Figure S17. Top 10 variables per SHAP value for cluster 6

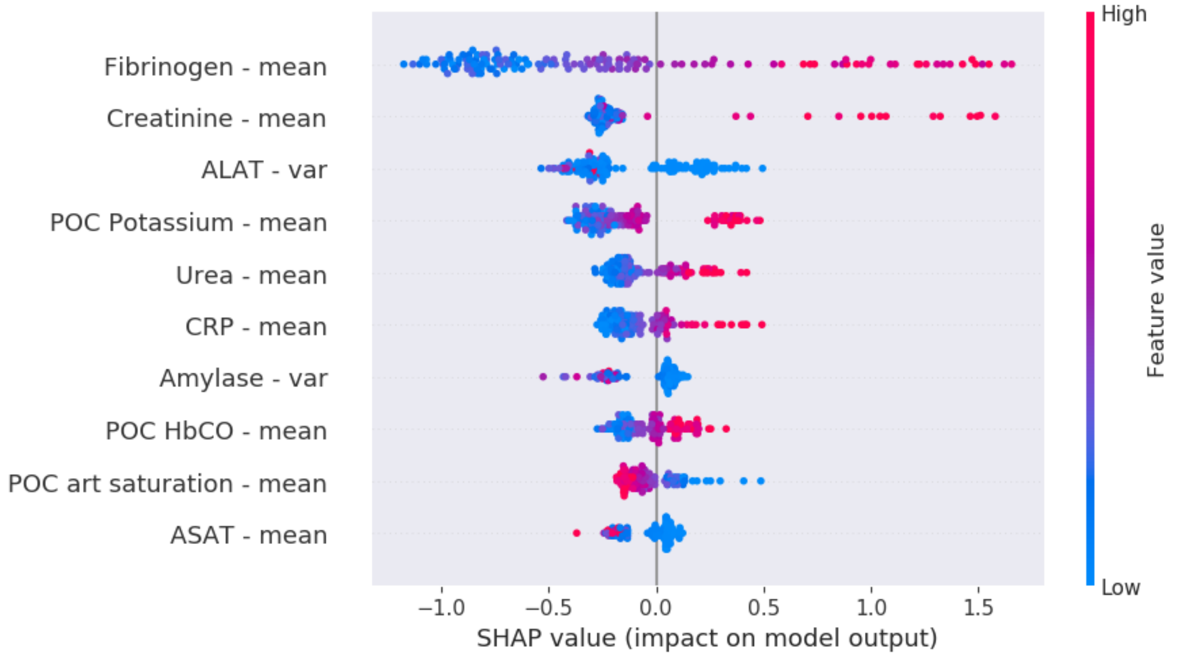

Supplementary methods

Dynamic time warping

For sequential multivariate time series to be usable for clustering, data have to be adequately pre-processed using other algorithms. One algorithm often used for pre-processing of (multivariate) time series is Dynamic Time Warping (DTW). Dynamic time warping defines a distance between each multivariate time series, and subsequently use algorithms that learn to predict classes or determine clusters based on distances between examples [1,2]. The DTW distance between two time series is calculated by determining the cost of aligning each time point in the two sequences, also taking into account the previous time steps. This is described by the following equation:

$$D(A_i, B_j) = \delta(a_i, b_j) + min \begin{Bmatrix} D(A_{i-1}, B_{j-1}) \\ D(A_i, B_{j-1}) \\ D(A_{i-1}, B_j) \end{Bmatrix}$$

Where A and B are the time series, Ai denotes the series up to time point i, whereas ai is the value of the time series at i. The distance function δ is defined as the Euclidean distance between two values at time points i and j. As can be seen from the formula, calculating the DTW distance is a recursive function which constructs a similarity matrix with the distances between all the time steps. The optimal path through this matrix (i.e. the smallest distance in total) is taken to be the alignment, and the distance between the two time series is defined as the total distance of the optimal path. In this study, the DTW distances for the multivariate time series were calculated as the sum of the distances between the univariate sequences.

Deep embedded clustering

Deep embedding clustering (DEC) algorithms utilize autoencoder neural networks to learn a certain representation of the data, and then use this representation to form clusters [3]. The DEC model developed in this study combined a multilayer perceptron (MLP) autoencoder with one hidden layer and a custom clustering layer utilizing k-means clustering algorithm. An overview of the algorithm made by Xie et al. (2016) can be seen below.

Figure S18. Schematic representation of the DEC algorithm

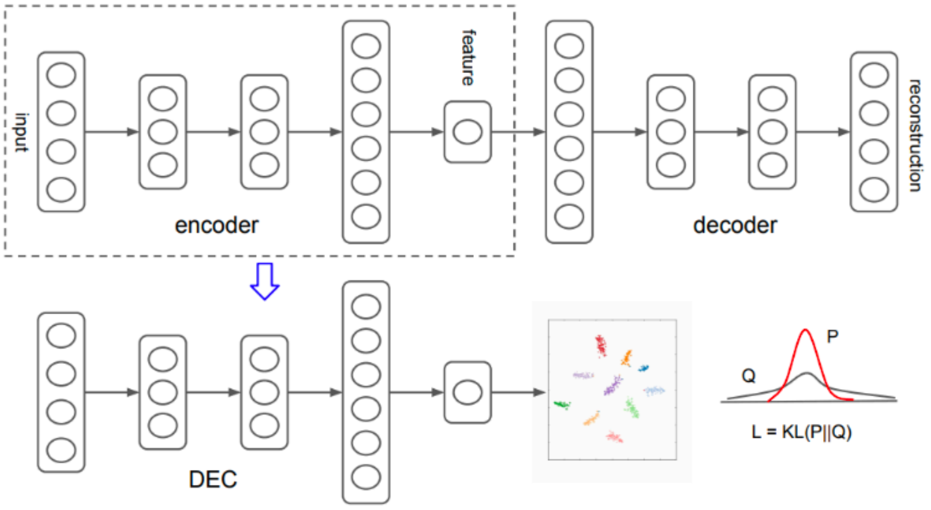

The clustering layer works as follows: the reconstructed features created by MLP autoencoder are converted to cluster label probability represented by Student's t-distribution. The clustering layer weights represent the cluster centroids and are initialized using k-means algorithm.

In order to improve cluster purity, a centroid-based target distribution was constructed by squaring the encoded vectors and normalizing them by frequency per cluster. Finally, the algorithm was trained to minimize Kullback–Leibler divergence loss for a maximum of 8000 iterations with 0.01 tolerance threshold.

Model	Parameters	Value
MLP autoencoder	Epochs	500
	Batch size	64
	Hidden layers (encoder and decoder)	1
	Units in hidden layer	64
	Units in encoding layer	8

	Dropout	0.4
	L1 regularizer	0.0005
DEC clustering layer	Batch size	256
	Stochastic gradient descent - learning rate	0.01
	Stochastic gradient descent - momentum	0.9

Supplementary references

1. Williams C, Rasmussen CE (2006). Gaussian processes for machine learning, volume 2. MIT Press Cambridge, MA.
2. Petitjean P, Ketterlin A, Gancarski P (2011). A global averaging method for dynamic time warping, with applications to clustering. Pattern Recognition 44(3):678–693.
3. Xie J, Girshick R, Farhadi A (2016). Unsu- pervised deep embedding for clustering analysis. In International conference on machine learning 478–487.
4. Rousseeuw PJ (1987) Silhouettes: A graphical aid to the interpretation and validation of cluster analysis. Journal of Computational and Applied Mathematics 20:53-65. doi: 10.1016/0377-0427(87)90125-7
5. Jain AK (2010) Data clustering: 50 years beyond *K*-means. Pattern Recognit Lett 31(8):651–666. Doi: 10.1016/j.patrec.2009.09.011
6. Handl J, Knowles J, Kell DB (2005) Computational cluster validation in post-genomic data analysis. Bioinformatics 21(15):3201-3212. Doi: 10.1093/bioinformatics/bti517
7. Hennig C (2007) Cluster-wise assessment of cluster stability. Comput Stat Data Analysis 52:258–71. https://doi.org/10.1016/j.csda.2006.11.025
